# Longitudinal genomic surveillance of SARS-CoV-2 in a university microcosm reflects global evolutionary trends

**DOI:** 10.1128/spectrum.00860-26

**Published:** 2026-06-15

**Authors:** Sankar Prasad Chaki, Melissa M. Kahl-McDonagh, Benjamin W. Neuman, Loni A. Taylor, Rebecca S. B. Fischer, Blake M. Hanson, Marlisa S. Hardy, Jo Ann Culpepper, Eric Boerwinkle, Kurt A. Zuelke

**Affiliations:** 1 Global Health Research Complex, Division of Research, Texas A&M University 14736, College Station, Texas, USA; 2 Department of Biological Sciences, Texas A&M University 14736, College Station, Texas, USA; 3 Epidemiology, Biostatistics, Texas A&M School of Public Health 26515, College Station, Texas, USA; 4 School of Public Health, University of Texas Health Science Center at Houston 49219, Houston, Texas, USA; 5 University Health Services, Texas A&M University 14736, College Station, Texas, USA; Barnard College, Columbia University, Biology, New York, New York, USA

**Keywords:** SARS-CoV-2, genomic surveillance, viral evolution, clades, lineages, variants, phylogenetics, epidemiologic, university community, public health

## Abstract

SARS-CoV-2 transmission has declined, yet ongoing viral evolution continues to pose public health challenges. This study conducted long-term (2020–2024) genomic surveillance within a large and globally diverse university community. Viral genomes obtained from clinical (*n* = 1,786) and surveillance (*n* = 888) testing samples were classified under clade, lineage, and variant systems, and compared with global genomic data sets. Local patterns broadly paralleled global evolutionary trends, including the phased emergence of major variants, episodic introductions of new lineages, and shifts in dominant clades. Lineage diversity peaked during fall semesters and was highest among undergraduate-aged individuals, with similar distributions across sexes. Phylogenetic analyses revealed transmission patterns characteristic of semi-contained, high-contact environments. These findings illustrate how large university communities can serve as microcosms of broader SARS-CoV-2 evolution. They also demonstrate the value of targeted genomic surveillance in generating epidemiologic intelligence to support early detection, track viral diversification, and inform public health preparedness.

**IMPORTANCE:**

This study highlights the public health and epidemiological value of academic laboratories and campus‑based testing programs in variant monitoring, outbreak detection, and pandemic preparedness. It also demonstrates how locally generated genomic data can effectively complement national and global surveillance networks.

## INTRODUCTION

SARS-CoV-2, the causative agent of the COVID-19 pandemic, resulted in 1.2 million deaths in the United States and 7.1 million deaths globally as of February 2026 (1, 2). U.S. universities responded to the COVID-19 pandemic in a variety of ways, balancing the need for public health safety while maintaining educational continuity (3–5). As the pandemic progressed, many colleges and universities adopted hybrid models, offering a mix of in-person and online classes to maintain some level of face-to-face interaction while minimizing health risks (6). At the same time, many also developed and implemented various SARS-CoV-2 testing strategies to address their individual situations (7–11). At some institutions, students, faculty, and/or staff who tested positive for SARS-CoV-2 were to isolate away from others, while exposed persons were asked to quarantine (12, 13).

Texas A&M University, which is one of the nation’s largest in-person campuses, implemented extensive laboratory testing as part of its disease control strategy, including on-demand clinical testing and surveillance testing among faculty, staff, and students to monitor infection trends and reveal opportunities for exposure prevention (14). This approach helped identify and isolate cases quickly, ultimately helping to reduce transmission and contain spread within the campus community through targeted prevention measures. Leveraging this multi-pronged testing strategy, the university played a vital role in SARS-CoV-2 variant surveillance through whole-genome sequencing, making it possible to monitor viral evolution, track emerging variants, and inform public health responses. Beginning in 2020, we assessed SARS-CoV-2 variants over a 4-year period through whole-genome sequencing and receptor-binding motif-targeted Sanger sequencing (7, 15) on specimens obtained from our university testing program. We identified genomic changes and variant trends over time, contributing to a deeper understanding of viral dynamics in a localized setting. Our approach involved systematic sample collection, RNA extraction, library preparation, and next-generation sequencing, followed by bioinformatics analysis to classify variants, submitting sequences to the Global Initiative on Sharing All Influenza Data (GISAID) and the U.S. Centers for Disease Control and Prevention (CDC).

Although numerous SARS-CoV-2 genomic surveillance studies have characterized viral evolution, most efforts were retrospective, limited in duration, or focused on broad regional or national trends. Distinct from this prior work, our study provides real-time sequencing with a coordinated institutional public health response during the early pandemic, followed by longitudinal variant monitoring. This continuity in monitoring enabled continuous actionable reporting to support campus-level mitigation strategies and contributed critical data to national and global genomic surveillance systems.

## MATERIALS AND METHODS

### Data collection and processing

Sequences for this SARS-CoV-2 analysis originated from clinical and non-clinical laboratory specimens received in our laboratory. Clinical specimens (nasal swabs in virus transport media) were derived from clinical sites, primarily from university health services. Non-clinical specimens (saliva) were derived from university-mandated surveillance efforts (16) and on-demand self-screening non-clinical testing sites on campus. All sequences were submitted to GISAID. For this analysis, data were downloaded directly from GISAID using filters for submissions by our site during the dates from October 2020 to October 2024, which included metadata (age and sex of the individual from whom the specimen was sourced).

### Viral RNA extraction

Viral RNA was extracted from saliva and nasal swab samples (17, 18) using the Quick-DNA/RNA Viral MagBead Kit (Zymo Research, USA) and the KingFisher Flex automated system (Thermo Fisher Scientific, USA), following the manufacturer’s protocol with minor modifications. Each 300 μL sample was mixed with 100 μL DNA/RNA Shield (R1200, Zymo Research, USA) and 5 μL proteinase K (20 mg/mL)and incubated for 15 min at room temperature. Sample lysis was performed with 600 μL RNA lysis buffer and 20 μL MagBinding Beads (D4100-2-12, Zymo Research, USA). RNA was treated with DNase I (Zymo Research, USA), eluted in 50 μL nuclease-free water, and stored at −80°C.

### RT-qPCR screening for sequencing suitability

RT-qPCR was performed using the CDC N1 primer/probe set (CDC N1-F, CDC N1-R, and FAM-probe) (19) and the Luna Universal Probe One-Step RT-qPCR Kit (E3006, NEB, USA) on a BIORAD CFX96 thermocycler, following previously described protocols (7). Each 20 μL reaction included 13 μL master mix and 7 μL sample. Cycling conditions were 55°C for 10 min (cDNA synthesis), 95°C for 1 min, followed by 41 cycles of 95°C for 10 s and 60°C for 30 s. Data were analyzed using Bio-Rad CFX Maestro and Excel. Samples with CT values <33 were prioritized for sequencing. Following RT-qPCR screening, a subset of positive samples with CT values <33 was prioritized for whole-genome sequencing library preparation and sequencing.

### SARS-CoV-2 cDNA synthesis, library preparation, and whole-genome sequencing

cDNA was synthesized from purified RNA using the SuperScript IV system (Invitrogen, CA, USA). The reaction included random hexamers, dNTPs, and reverse transcription mix and was run on a BIORAD CFX96 thermocycler. The resulting cDNA was used for library preparation with the xGen SARS-CoV-2 amplicon panel kit from Integrated DNA Technology (previously from Swift) (20, 21), involving multiplex PCR, magnetic bead purification, and index adapter ligation. Libraries were quantified using Qubit and TapeStation, then pooled for sequencing on Illumina NextSeq 550/2000 platforms. Each sample yielded~1million reads, which were mapped and assembled using BWA. Sequences were submitted to GISAID.

### Bioinformatics analysis of SARS-CoV-2 whole-genome sequencing data

Raw sequencing reads were first subjected to quality assessment using FastQC (v.0.11.9) to evaluate base quality scores, GC content, and sequence duplication levels. Sequencing adapters and low-quality bases were trimmed using Trim Galore (v.0.6.6), which incorporates Cutadapt (v.2.10) for adapter removal. High-quality trimmed reads were then aligned to the SARS-CoV-2 reference genome (NC_045512.2) using BWA-MEM (v.0.7.17). Variant calling was performed using bcftools (v.1.11) to identify single-nucleotide polymorphisms and insertions/deletions (indels). Pangolin (v.4.3) (GISAID) and NextClade (v.2.14.1) were employed to assign SARS-CoV-2 clades and lineages.

### Clade, lineage, and variant classification

SARS-CoV-2 sequences were classified using the GISAID system, which identifies major phylogenetic clades based on marker mutations (22), and Pango lineages to capture finer-scale evolutionary and regional transmission patterns (23). WHO-designated variants of concern (VOCs), variants of interest (VOIs), and variants under monitoring (VUMs) were assigned to lineages with increased transmissibility, immune escape, or public health impact, as integrated into GISAID’s analysis pipeline (24, 25). Distribution patterns were analyzed and visualized using Microsoft Excel and GraphPad Prism 9.

### Clade subclassification

GISAID’s clade classification system, while foundational for tracking SARS-CoV-2 evolution, terminates at the GRA level, limiting the ability to distinguish among the many sub-lineages of the Omicron variant. To overcome this limitation, we developed an extended clade subclassification scheme that builds upon the original GISAID clades by incorporating major SARS-CoV-2 lineage prefixes.

### Global comparison

Global data were also retrieved from the GISAID EpiCoV database using filters for host (human) and collection dates from October 2020 to October 2024. Due to data set size limitations, quantitative outputs were recorded directly in Excel spreadsheets rather than downloading complete data sets. For lineage ranking, each of the locally identified lineages was queried individually, and the corresponding global sequence counts were recorded for secondary analysis. At the time of analysis, the database contained 16,871,076 sequences, of which 11,542,649 corresponded to these lineages. To identify variant arrivals, we restricted the search to human samples within the same date range and used GISAID’s dropdown selections for variants (VOCs, VOIs, and VUMs). In total, 16,036,225 records were sorted by sample collection date to determine the earliest cases. These were then compared with our local data to calculate arrival gaps. Of the 25 variant groups listed in the GISAID dropdown menu, 24 were present during the study period and were included in the comparison. Global clade comparisons (*N* = 16,733,813) were performed using the same human sample and date range filters. All 12 clade options available in the GISAID dropdown menu were included in the analysis.

### Phylogenetic analysis and tree construction

Phylogenetic analysis was conducted to examine evolutionary relationships among distinct SARS-CoV-2 Pango lineages identified over the analysis period. Consensus genomes annotated via GISAID were aligned and analyzed using CLC Sequence Viewer 8.0 (Qiagen). Phylogenetic trees were constructed using the Neighbor-Joining method with the Kimura 80 (K80) substitution model. Tree robustness was assessed with 1,000 bootstrap replicates, considering values ≥70% as strong support. The tree was rooted with the Wuhan-Hu-1 reference genome (NC_045512.2) to establish an evolutionary baseline. Visualization and annotation were performed using CLC Sequence Viewer and FigTree (v.1.4.4).

### Statistical analysis

Data organization, cleaning, and statistical analyses were performed using Microsoft Excel and GraphPad Prism (v.9.5.1). Where relevant, 95% confidence intervals were calculated to support statistical estimates. *P*-values <0.05 were considered statistically significant. Lineage diversity for the age group and sex was estimated using the non-parametric Chao estimator to adjust for unseen taxa (26). Specifically, we noted which lineages were observed only once in the sequence data (singletons, a) and lineages observed twice (doubletons, b). The total number of lineages observed (S_obs_) was also reported by age and sex.

## RESULTS

A total of 2,674 sequences spanning 4 years of surveillance, October 2020 to October 2024, were included in this analysis (888 from surveillance and screening activities and 1,786 from clinical encounters). Most specimens were from the age group (18–30 years), and a small proportion came from younger or older individuals (Table S1).

### Monthly SARS-CoV-2 clade diversity in the university community

**Fig 1:**
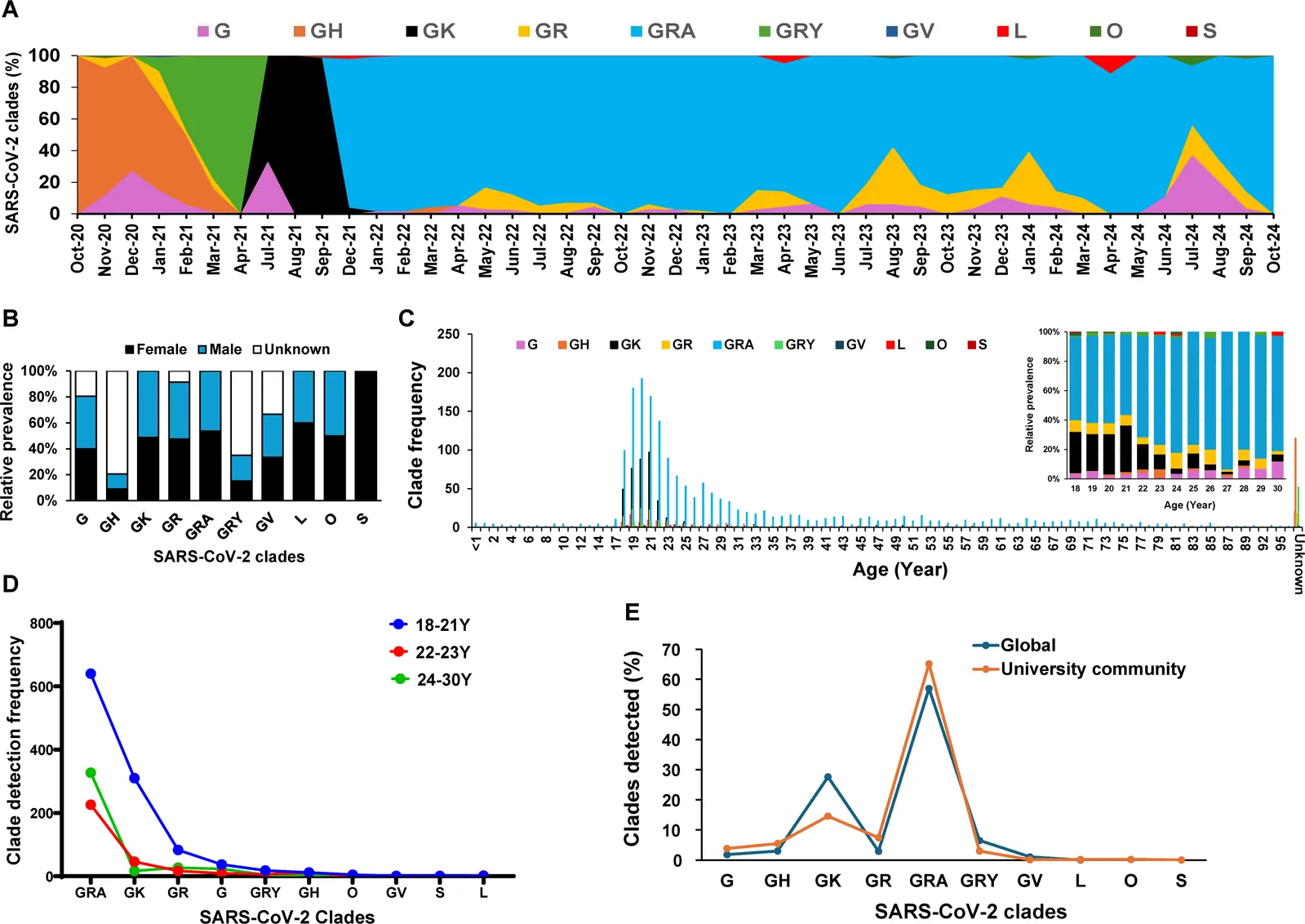
Diversity and temporal distribution of SARS-CoV-2 clades in the university community (October 2020–October 2024). (**A**)Area chart displaying monthly percentages of SARS-CoV-2 clade detection over 4 years. (**B**)Stacked column chart illustrating the sex-based relative detection of SARS-CoV-2 clades. (**C**)Column chart showing the age-based detection frequency of various SARS-CoV-2 clades noticed in the community. (**D**)Line graph comparing the detection of SARS-CoV-2 clades in academic age groups. (**E**)Line chart comparing the detection of SARS-CoV-2 clades in the university community with global trends.

In this community, 10 of 12 GISAID clades were observed in circulation in varying proportions. Monthly clade diversity ranged from one to six clades, with dynamic shifts in dominance over time. Early dominance by clade GH (up to 80.6% in Nov 2020) was later replaced by clade GRY, which peaked at 100% in April 2021. Clade GK then surged, reaching 100% in August 2021 but declining sharply by December 2021. It was replaced by clade GRA, which dominated from late 2021 to 2024. Although GRA dominated throughout, this period included brief, recurrent re-emergence of clades GR and G. Clades GV, L, S, and O were consistently rare, ranging from 0.35% to 6.25%. Clades V and GKA were not observed in our analysis of this community (Fig. 1A).

### Sex-based distribution of SARS-CoV-2 clades

Clade distribution was similar between females and males for major clades (G, GK, GR, GRA). However, clades GH (79%) and GRY (65%) were frequently observed in individuals whose sex was not reported, and rare clades appeared evenly distributed. Only one case of clade S was identified in a female, reflected in the skewed distribution of 100% (Fig. 1B). One-way ANOVA revealed no significant differences in clade proportions by sex (*P* = 0.0896).

### Age-based distribution of SARS-CoV-2 clades

Ten clades appeared across age groups, with GRA dominant in all groups, followed by GK, GR, and G (Fig. 1C). GK was more common in ages 18–23 years, becoming less common in ages 24–30 (Fig. 1C inset). Other clades, such as S, L, GV, and O, appeared only in a few cases. Clade proportions varied significantly within each age category (*P* < 0.0001), but not between categories (*P* = 0.9626), with GRA being the most common across groups (Fig. 1D).

### Distribution of SARS-CoV-2 clades vs the global context

A comparison of SARS-CoV-2 clade distributions between the global data set and the university community from October 2020 to October 2024 revealed both similarities and differences (Fig. 1E). The GRA clade was the most dominant in both data sets, comprising 57% globally and an even higher 65.2% within the university population. GK was the next dominant clade identified both in the university community (14.6%) and in the global (27.6%) data set. Clades GR, GH, and G were more common, while GK and GRY were observed at lower proportions in the university community. A temporal correlation was observed between local and global clade proportions (*r* = 0.96, *P* < 0.0001).

### Clade subclassification

Lineage-based subclassification revealed variable numbers of subclades across clades identified in the community, with GRA showing the highest diversity (Table 1). This refined classification enables more accurate differentiationbetween genomes within the same clade that emerged at different times in the community, for example, distinguishing G and GR clade genomes from 2020 versus those from 2024, thereby improving temporal and epidemiological resolution in genomic surveillance.

**TABLE 1:** Newly assigned subclades based on major SARS-CoV-2 lineage prefix^*a*^

GISAID clade	Newly assigned subclades
G	G-AY, G-B, G-BA, G-BQ, G-EG, G-FL, G-JN, G-XBB
GH	GH-B
GK	GK-AY, GK-B
GR	GR-B, GR-BA, GR-BE, GR-BF, GR-BN, GR-EG, GR-FD, GR-FL, GR-HN, GR-HS, GR-HV, GR-HZ, GR-JD, GR-JN, GR-P, GR-R, GR-XBB
GRA	GRA-BA, GRA-BE, GRA-BF, GRA-BN, GRA-BQ, GRA-BU, GRA-CA, GRA-CH, RA-CK, GRA-EA, GRA-ED, GRA-EG, GRA-EY, GRA-FB, GRA-FL, GRA-FU, GRA-GE, GRA-GJ, GRA-GK, GRA-GN, GRA-HF, GRA-HH, GRA-HK, GRA-HN, GRA-HS, GRA-HV, GRA-HY, GRA-JD, GRA-JF, GRA-JG, GRA-JN, GRA-KP, GRA-LB, GRA-XAF, GRA-XAS, GRA-XBB, GRA-XBC, GRA-XBD, GRA-XCK, GRA-XEC, GRA-XW
GRY	GRY-B.1.1.7
GV	GV-AY, GV-B, GV-XBC
L	L-B
O	O-B, O-BA, O-JN
S	S-A

^*a*^ Note that a single Omicron sublineage was represented across multiple GISAID clades; therefore, subclades were reassigned by combining GISAID clade and lineage information.

### SARS-CoV-2 lineage diversity in the university community

**Fig 2:**
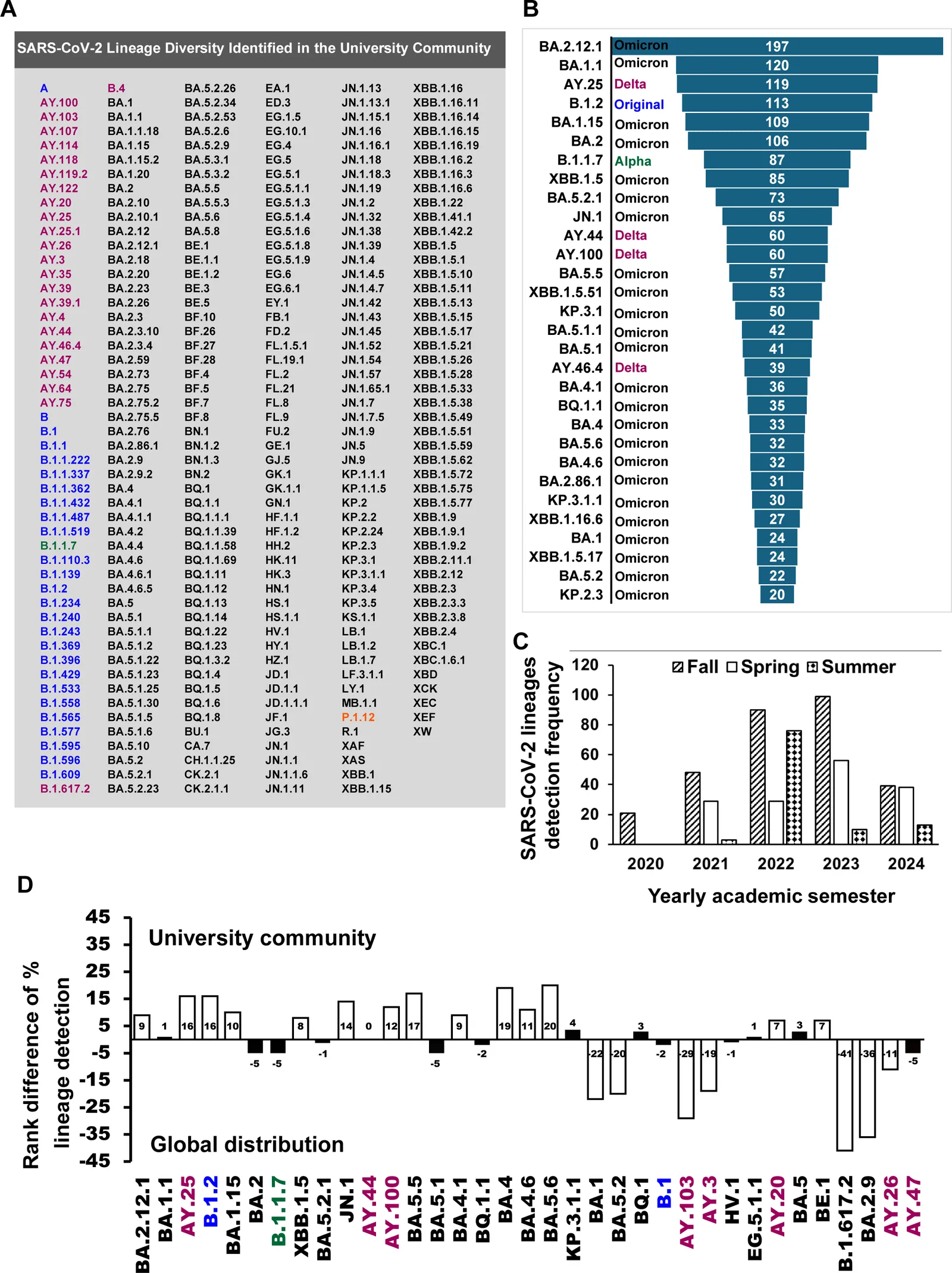
Diversity of SARS-CoV-2 lineages in the university community (October 2020–October 2024). Major lineages are color-coded as follows: A/B early sublineages (blue), Alpha (green), Gamma (orange), Delta (purple), and Omicron (black). (**A**)A total of 296 diversified SARS-CoV-2 lineages were identified in the university community over 4 years. (**B**)Funnel chart showing the top 30 most frequently identified SARS-CoV-2 lineages in the community. (**C**)Yearly academic semester-based lineage detection frequency. (**D**)Rank difference of 35 shared SARS-CoV-2 lineages among the top 50 detected in university and global data sets (*N* = 2,639 vs11.5M). Black bars indicate minimal ranked variation (0–5).

We identified the diversity of SARS-CoV-2 lineages by month over 45 months of genomic surveillance. Across all months, this totaled 551 lineage-month observations (Fig. S1A), representing 296 unique lineages (Fig. 2A). These included persistent and transient lineages, with several classified as VOCs, VOIs, or VUMs, according to WHO or CDC guidelines. The most frequently detected lineage was BA.2.12.1 (Omicron), followed by AY.25, AY.44, and AY.100 sub-lineages of the Delta variant, and B.1.1.7 (Alpha variant). Other notable Omicron sub-lineages included BA.1.1, BA.1.15, BA.2, BA.4.1, BA.5.1, BA.5.2.1, and BA.5.5, and recombinants like XBB.1.5, XBB.1.5.17, XBB.1.5.51, and XBB.1.16.6. Late emerging lineages such as BA.2.86.1, JN.1, KP.2.3, and KP.3.1.1 were among the top 30 detected in the community (Fig. 2B). The highest lineage detection (297/551) was observed during fall semesters, followed by spring (152) and summer (102), with summer 2022 showing an exception (Fig. 2C).

### Distribution of SARS-CoV-2 lineages vs the global context

A comparison of SARS-CoV-2 lineages observed in our specimens (*N* = 2,639 lineages) and the global data set (*N* = 11,542,649 lineages) revealed a complex pattern of both alignment with and deviation from global trends. Of the 50 most frequently detected SARS-CoV-2 lineages locally, 35 overlapped with the top 50 globally prevalent lineages. Notably, 14 of these shared lineages (black bars) exhibited minimal rank variation (difference of 0–5), suggesting consistent global and local circulation patterns (Fig. 2D). BA.2.12.1 was the most dominant locally (7.46%) but ranked 10th globally (2.60%). BA.1.1, another Omicron subvariant, ranked second in the community (4.55%) and was also one of the top lineages globally (9.41%, rank 3). Lineage AY.44 ranked 11th (2.3%) both locally and globally, reflecting strong consistency between local and international transmission dynamics for this variant. Other lineages, such as AY.25 (4.51%), B.1.2 (4.28%), and BA.1.15 (0.13%), had higher local prevalence, indicating localized transmission dynamics. The Pearson correlation between local and global detection percentages was 0.58 (*P* < 0.0001), indicating moderate alignment in lineage prevalence. However, the paired *t*-test showed no significant difference in mean detection percentages (*P* = 1.0), suggesting overall proportional balance despite individual lineage disparities.

### Temporal lineage richness

**Fig 3:**
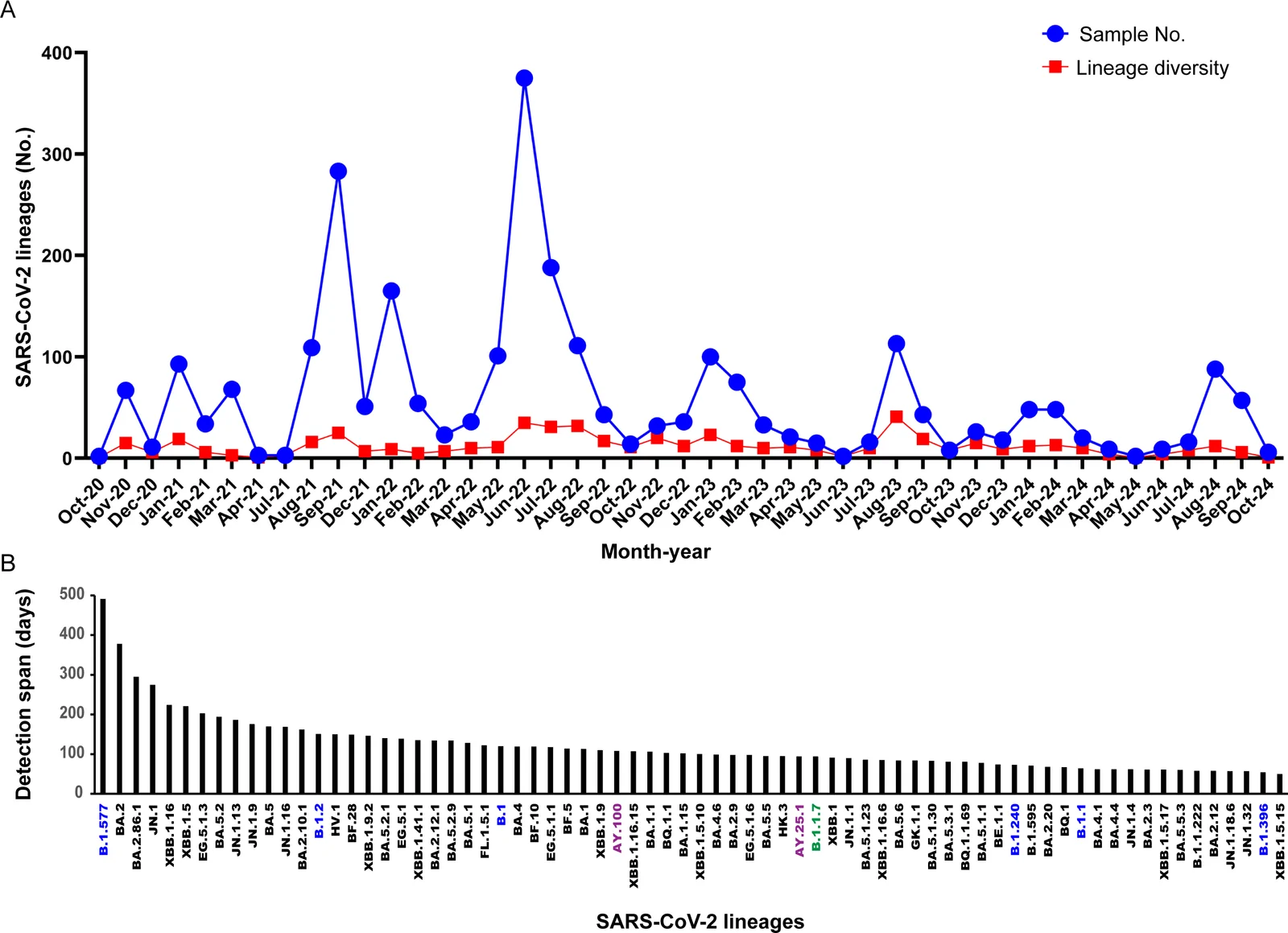
Monthly lineage diversity and duration of detection in the university community. (**A**)Monthly trends of SARS-CoV-2 lineage diversity relative to sample volume in the university community. (**B**)Bar chart showing the duration between the first and last detection (detection span ≥50days) of individual SARS-CoV-2 lineages in the community over the 4-year period. Major lineages are color-coded as follows: A/B early sublineages (blue), Alpha (green), Delta (purple), and Omicron (black).

Analysis of lineage diversity richness over time revealed lineage peaks (red line)**,**which were highly correlated (*r* = 0.84, *P* < 0.0001) with the number of samples tested/infection waves (blue line). Temporal diversity peaks (red line) occurred in Nov. 2020, Jan. 2021, Sept. 2021, June 2022, Nov. 2022, Jan. 2023, Aug. 2023, Nov. 2023, Feb. 2024, and Aug. 2024 (Fig. 3A).

### Span of lineage detection

We calculated the span of lineage detection as the time between the first and last observation of each lineage. Out of 296 diversified lineages we observed, 135 were detected only once in our analysis. Among other lineages, 37 lineages showed a detection span of≥100 days, and 72 lineages had a detection span of≥50 days (Fig. 3B; Fig. S2). The lineages observed for the longest detection span were B.1.577 (491 days), BA.2 (378 days), BA.2.86.1 (295 days), and JN.1 (275 days). Shorter detection spans were noticed for XBB.1.16.19, BF.8, and AY.47,ranging from7 to 12 days.

### Sex-based distribution of SARS-CoV-2 lineages

**Fig 4:**
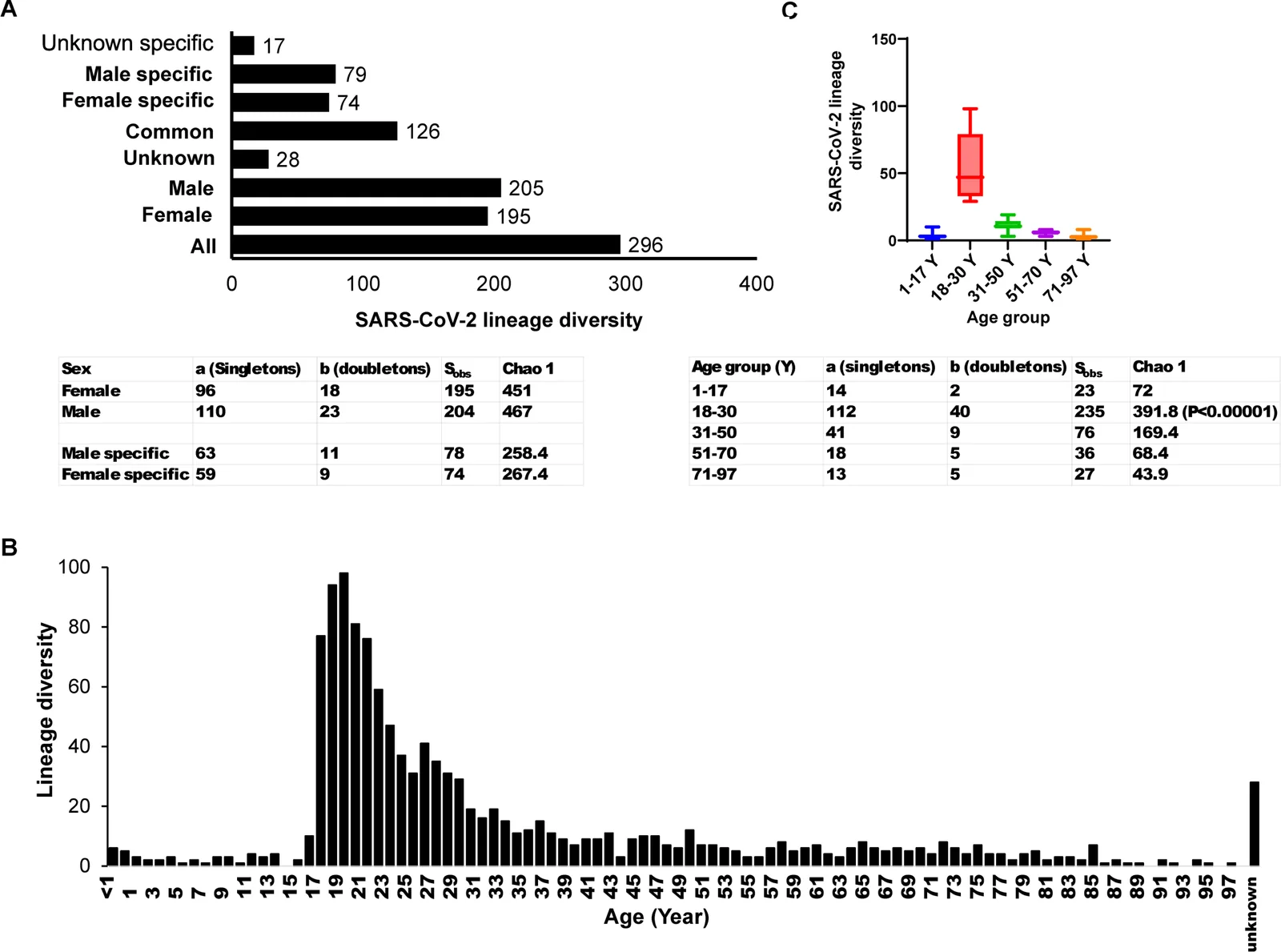
Sex- and age-based SARS-CoV-2 lineage detection in the university community. (**A**)Distribution of SARS-CoV-2 lineages among males, females, and individuals with unknown sex. Bottom panel shows Chao estimate of biodiversity in different sex groups. (**B**)Bar chart showing age-based detection of SARS-CoV-2 lineages in the university community. (**C**)Boxplot illustrating the quantitative distribution of SARS-CoV-2 lineages across different age groups. Bottom panel shows Chao estimate of biodiversity in different age groups.

Among the 296 lineages, 79 were observed only in males, 74 only in females, and 17 only in individuals of unknown sex. The remaining 126 lineages were observed in at least two sex categories including males, females, and unknown (Fig. S3). Overall, 205 lineages were found in males, 195 in females, and 28 in individuals with unspecified sex (Fig. 4A). Chao1 biodiversity estimates were comparable between females and males, as well as between female-specific and male-specific lineages (Fig. 4A, bottom panel).

### Age-based distribution of SARS-CoV-2 lineages

We identified the diversity of SARS-CoV-2 lineages across various ages; this totaled 1,203 lineage-age observations, reflecting overlaps where certain lineages occurred in multiple age groups (Fig. S4). Dominant lineages included BA.2.12.1, BA.2, BA.1.1, BA.5.5, BA.5.1.1, BA.1.15, BA.5.2.1, BA.4.1, BA.5.1, and BA.5.6 (Fig. 4B). Boxplots illustrated a clear peak in lineage diversity among young adults, particularly those in the 18–30 age range, with a broad interquartile range and several high-value outliers (Fig. 4C). The Chao1 biodiversity estimate for the student-age population (18–30) was nearly double that of all other age groups, and this difference was statistically significant (*P* < 0.00001) (Fig. 4C, bottom panel). The youngest individuals in this sub-population(18–21 years) demonstrated the highest lineage diversity (179), followed by the oldest within this age group (128) (Fig. S4).

### SARS-CoV-2 variants distribution in the community

**Fig 5:**
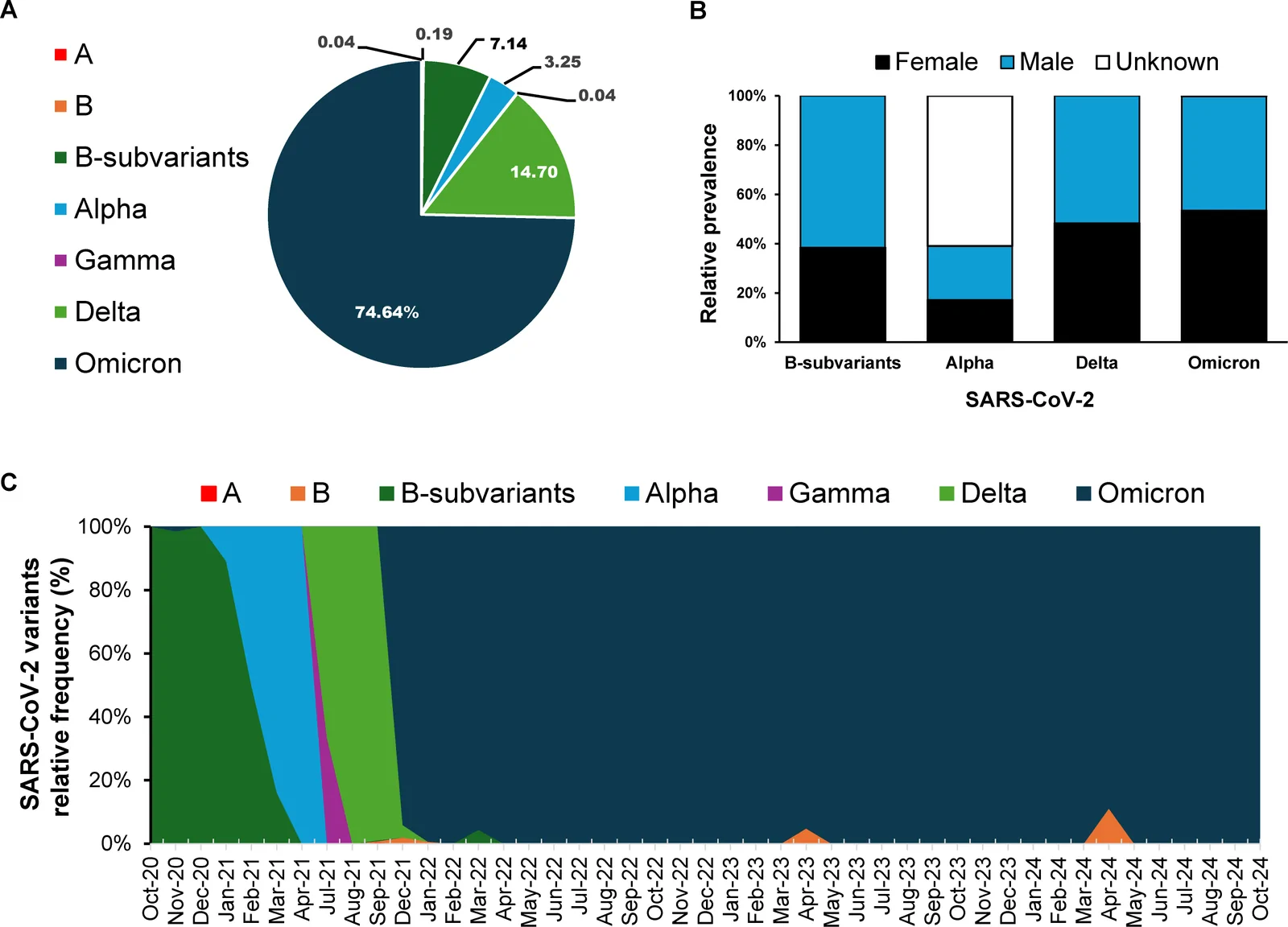
Variant-based characterization of SARS-CoV-2 in the university community. (**A**)Pie chart illustrates the cumulative detection of major SARS-CoV-2 variants identified in the community, with Omicron accounting for the majority of cases (74.64%). (**B**)Bar chart depicting the sex-based distribution of major SARS-CoV-2 variants circulating in the university community. (**C**)Monthly detection of major SARS-CoV-2 variants, showing the shift from early variants (Alpha, Gamma, and Delta) to the dominance of Omicron.

**Fig 6:**
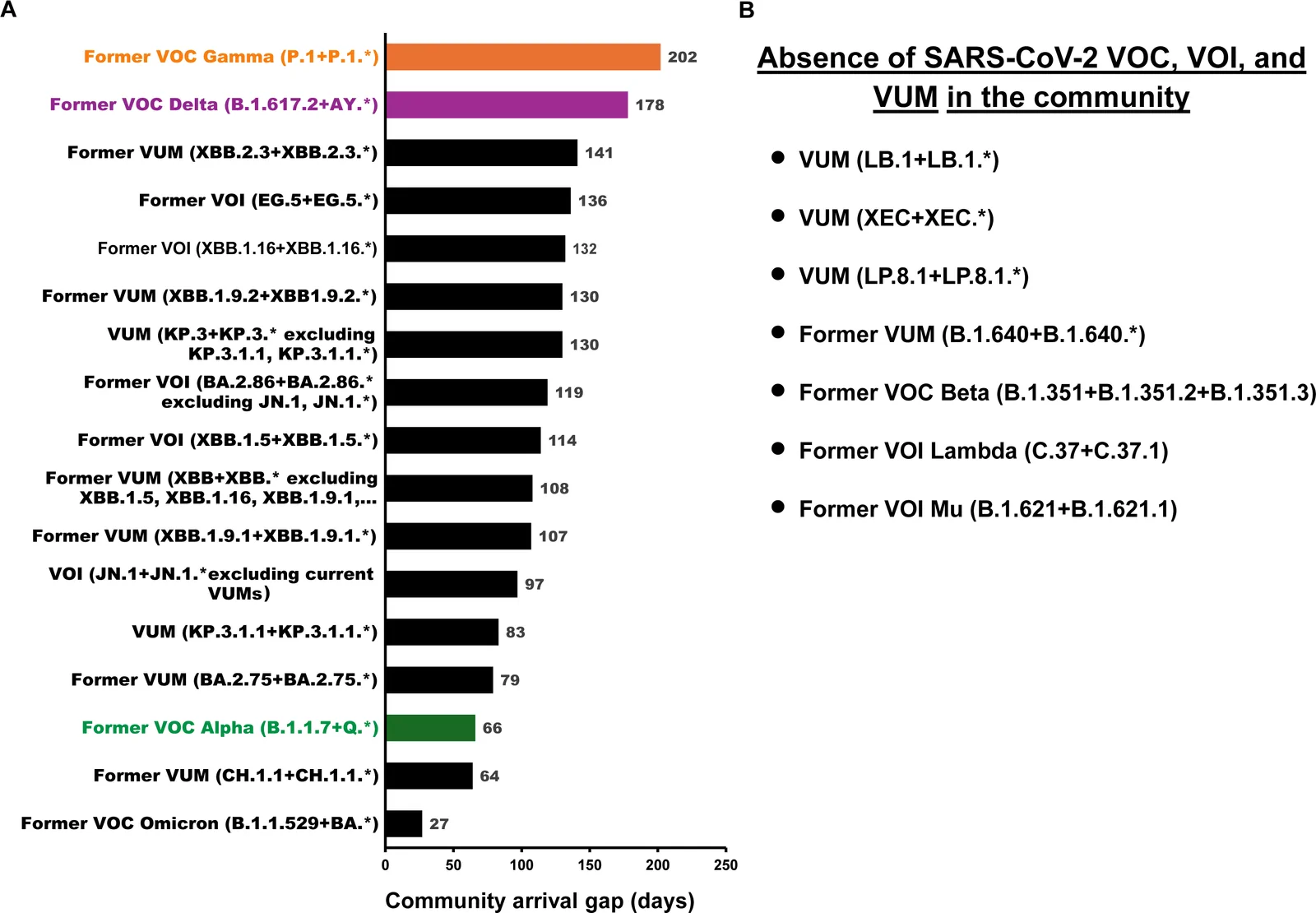
Lag between first detection of GISAID-listed categories of SARS-CoV-2 VOCs, VOIs, and VUMs in the university community. (**A**)The bar chart shows the number of days between the first identification of each category in the university community and the date it was first reported in the GISAID database. Major variants are colorcoded as follows: Alpha (green), Gamma (orange), Delta (purple), and Omicron (black). (**B**)Seven of the 24 categories of SARS-CoV-2 variants were absent in the community.

Over the 4-year surveillance period, the Omicron variant and its sub-lineages overwhelmingly dominated the SARS-CoV-2 landscape, accounting for 74.64% of all detected cases (Fig. 5A). In contrast, Delta was the second most frequent variant at 14.7%, followed by Bsubvariants (7.14%) and Alpha (3.25%). There was a similar proportion of Omicron, Delta, and Alpha major variants in females and males (Fig. 5B). When compiling temporal data on the emergence of SARS-CoV-2 variants (VOC, VOI, and VUM), we see that our data reflect a progression from early variants Alpha, Gamma, and Delta to dominance by Omicron. B subvariants were the predominant circulating variants during the early stages of the pandemic (2020), and as the pandemic progressed, Alpha became the dominant variant in early 2021, followed by the Delta variant later in the year. By late 2021 and early 2022, the Omicron variant rapidly emerged and dominated thereafter (Fig. 5C). Out of 24 GISAID-listed categories that persisted throughout the study period, 17 were also observed in our data (Fig. 6A), while 7 were not (Fig. 6B). The mean lag between global appearance and detection in our data was 113 days (SD = 42.4), ranging from a minimum of 27 days to a maximum of 202 days (median = 114 days).

### Phylogenetic tree of community-detected lineages

**Fig 7:**
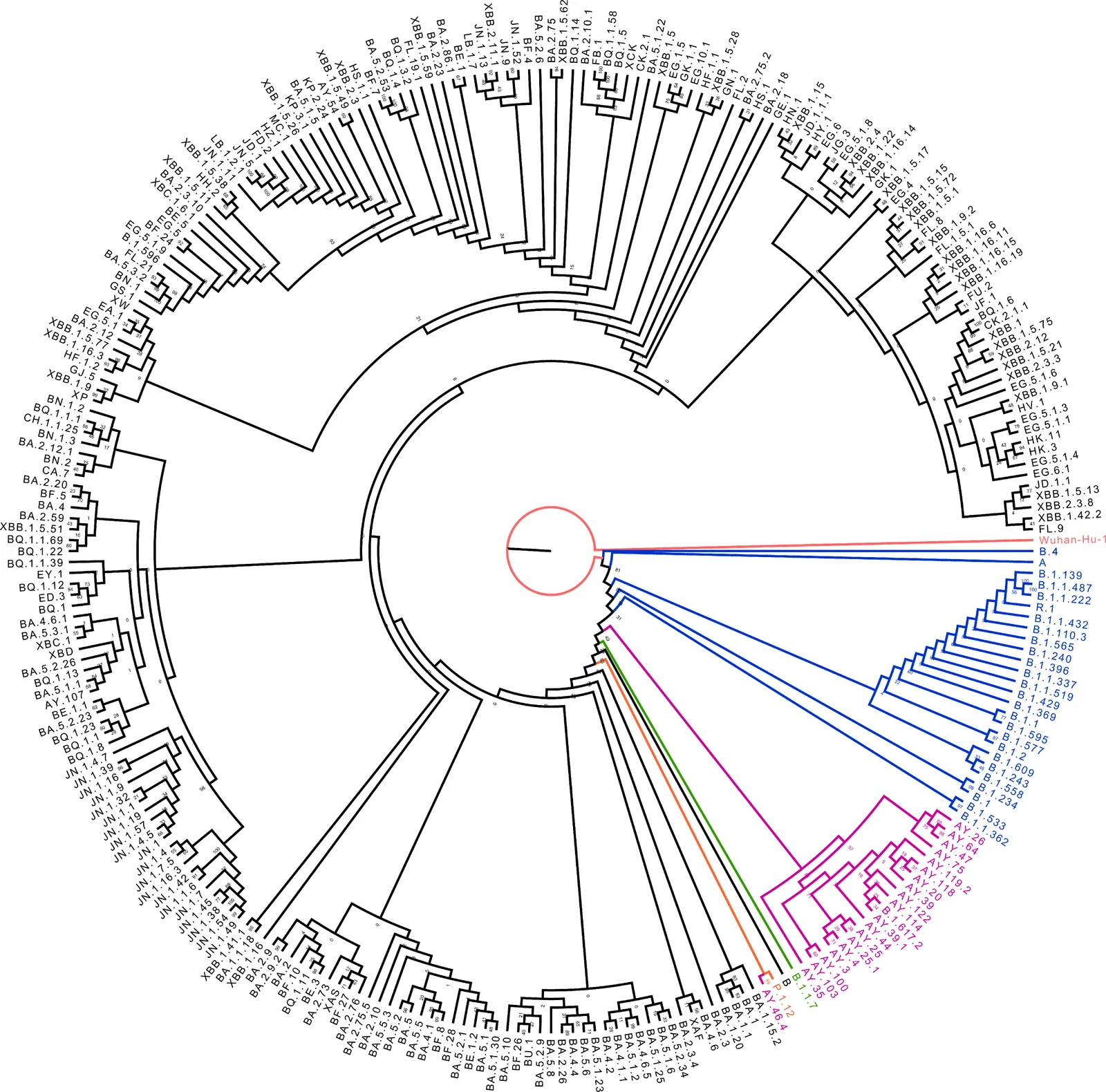
Phylogenetic tree illustration depicting the branching of SARS-CoV-2 lineages prevalent in the community. The tree was rooted with the ancestral sequence Wuhan-Hu-1 (NC_045512.2) and displayed the evolutionary emergence of diverse sub-lineages over time. Variants are color-coded as follows: early lineages (blue), Alpha (green), Gamma (orange), Delta (purple), and Omicron (black).

Phylogenetic analysis of 283 SARS-CoV-2 lineages reveals extensive diversification of SARS-CoV-2 since the initial Wuhan-Hu-1 isolate (Fig. 7). Early dominant lineages like B.1, B.1.1.7 (Alpha), and B.1.617.2 + AY.* (Delta) were replaced by diverse Omicron sub-lineages (BA.1–BA.5) and recombinants such as XBB.1.5, XBB.1.16, and XBB.2.3. Late emerging variants like JN.1, EG.5, and KP.3.1 reflect ongoing adaptation, while rare lineages (e.g., HK.3, HS.1.1) suggest localized transmission. The unrooted cladogram, visualized in a circular format, shows radial diversification from the Wuhan-Hu-1 ancestor, with Omicron sub-lineages (e.g., XBB.1.5.x, EG.5.x, JN.1.x) clustering at the periphery, indicating rapid mutation and spread. Well-supported monophyletic groups (e.g., BQ.1.x, BA.5.x) and long branches for new variants (e.g., KP.3.1.1, FD.2) suggest recent divergence. Classical lineages like B.1.1.7 (Alpha), B.1.617.2 (Delta), and P.1.12 (Gamma) are located closer to the root, indicating their early emergence and now lower detection in the current viral landscape.

## DISCUSSION

This 4-year genomic surveillance study provides insight into SARS-CoV-2 evolution and transmission dynamics within a large, globally diverse university community. By analyzing clades, lineages, variants, and phylogenetic relationships, we demonstrate that university settings can serve as effective microcosms for tracking global viral trends. However, some differences suggest that localized transmission dynamics within the university community do not always mirror the global context (27–29).

A temporal map of SARS-CoV-2 clades observed in samples from our community reflected what has already been reported about the trajectory of virus evolution. The transition from GH dominance in late 2020 to GRY (Alpha) in early 2021, followed by GK (Delta) in mid-2021, and ultimately GRA (Omicron) from late 2021 onward, mirrors global patterns (30–35). The sustained prevalence of GRA through 2023 and its diversification in 2024, including the reappearance of clade G, suggests ongoing viral adaptation under population immunity and environmental pressures. We do note that analysis of publicly available sequence data presents some challenges. We noted that GISAID assigned the G clade designation to both some older and newer lineages, making differentiation challenging, necessitating a newly devised subclassification system (Table 1) to resolve ambiguities in GISAID’s clade assignments. The standard GISAID clade system provides a broad overview of viral evolution. While some clades, such as GH (orange) and GRY (green), aligned with a single SARS-CoV-2 lineage during specific periods, others, including G (pink), GR (yellow), and GRA (blue), appeared across multiple years (Fig. 1A) despite shifts in circulating sub-lineages. This made it difficult to interpret long, continuous clade patterns, as the same clade could represent different sub-lineages over time. The subclassification system allows for a more granular approach to distinguish changes in clades over time.

Clade distributions in our analysis corroborated what has been reported worldwide, suggesting the potential utility of a similar diverse and large community as a model for large-scale variant surveillance. GRA was dominant across all population sub-groups in our community, reiterating its widespread transmission (36). Statistical comparisons between global and university clade distributions revealed a strong positive correlation (*r* = 0.96, *P* < 0.0001), an expected finding that demonstrates suitability for a large university community, such as this, in a role as a surveillance model for monitoring global viral variants.

We also examined SARS-CoV-2 sub-lineages to gain a more granular understanding of viral evolution in our setting. In so doing, we identified 296 distinct Pango (23) lineages across 551 lineage-month observations, understanding the evolutionary potential and adaptability of the virus. Thirty of our most frequent lineages are also aligned with top global lineages. Notably, local dominance of BA.2.12.1, AY.25, B.1.2, JN.1, and BA.5.5, during a global dominance of BA.2, B.1.1.7, B.1.617.2, and AY.4,suggests that unspecified factors in our community differ substantially from the global context. Such factors might include host immunity, vaccine diversity, vaccination uptake, ecological parameters, human behaviors, and other disease determinants across diverse populations. Factors unique to younger individuals, college campuses, and other congregate populations that may drive viral lineage composition would be useful to understand for future monitoring of viral evolution. Although we identified a single case of the XEC lineage in our community, we were unable to monitor the XFG, LP.8.1, and NB.1.8.1 SARS-CoV-2 lineages that predominated during 2025 and early 2026 (37) because of limited funding support.

As expected, we observed major VOCs Alpha, Gamma, Delta, and Omicron (25) in our community, with Omicron as the most frequent overall (74.64%). Delta (14.70%) dominated mid-pandemic, associated with increased severity and transmission. Alpha (3.25%) and Gamma (0.19%) were rare, and Beta was undetected, suggesting limited community transmission. The progression from Alpha to Omicron we observed also mirrors global epidemiological shifts (38). These variants were distributed across both sexes and all age groups, demonstrating that in our community, as in others, they had widespread transmission throughout the community. Our analysis revealed extensive phylogenetic diversification in our community strains from the Wuhan-Hu-1 reference strain (39).

The temporal dynamics of important variants of public health concern (VOC, VOI, and VUM) in the university community broadly mirror what has been observed globally, but with some temporal lag. As such, our local patterns may appear as a delayed reflection of the global scenario. Temporal diversity peaks in mid-2022 and August 2023 coincided with Omicron subvariant waves and possible new introductions. A decline in diversity post-May 2024 reflects viral stabilization, perhaps due to increasing population immunity. Semester-based analysis showed fall as the peak period for lineage diversity, likely driven by academic congregation following a summer period of geographic dispersion. We observed lineages that only appeared in one sex or the other, namely 79 male-specific and 74 female-specific observations, suggesting there may be subtle demographic determinants for specific viral strains (40–44).

To correct sampling biases in the number of unique SARS-CoV-2 lineages observed in different sex and age groups, we used a Chao estimate of biodiversity (Fig. 4). These diversity estimates did not differ meaningfully between females and males, nor among female-specific and male-specific lineages. The Chao estimate of biodiversity in the student-age population (18–30 years) was about twice that of all other age groups, which in our community include mainly faculty, staff, and, to a lesser extent, family members. As it appears many strains observed in this primarily student-age population did not spill into other age groups. While interesting, it is not unexpected that transmission, including outbreaks, occurred in isolation within this age population. In part, the university’s SARS-CoV-2 surveillance and screening program was intended to identify and track clusters of infection, and outbreaks occurred on campus and in the campus community. The epidemiologic isolation of this group from other age groups likely reflects a higher frequency or intensity of interactions conducive to SARS-CoV-2 circulation within the group, but a lower degree of interaction with other age groups. Although 74% of our samples were from the age group (18–30 years), a small proportion came from older adults (23.1%, 31–97 years), with very few from children or adolescents (2.3%,<18 years), so we must also consider that greater lineage diversity in the youngest adult age group could be a result of their over-representation in our community.

Amid challenges inherent to global surveillance systems, including limited infrastructure and capacity for widespread genomic sequencing, compounded by added challenges during a pandemic such as barriers to testing, underreporting, stretched infrastructure, and supply-chain failures, genomic monitoring in high-contact, semi-contained settings can provide a valuable lens through which to gain epidemiologic intelligence. Our study also underscores the crucial role of academic laboratories in public health surveillance, not only on a local scale but within the greater surveillance framework, to supplement and complement systems that inform effective public health strategies.

A notable strength of our study is the availability of sequences from both active and passive epidemiologic surveillance, affording the opportunity for a broader understanding of SARS-CoV-2 viral ecology beyond what presents at the clinical interface. The nature of the testing done at our site reflects the temporal fluctuations imposed by several rounds of university-mandated surveillance testing, gaps in testing (due to university holidays), and a lower frequency of sequences (owed to decreasing sample collection) in 2023–2024. Future efforts should incorporate a more systematic sampling and develop already established partnerships with local hospitals and clinics to ensure sample availability and enhance demographic diversity.

## Supplementary Material

Reviewer comments

## Data Availability

All genome sequences and associated metadata in this data set are published in GISAID’s EpiCoV database. To view the contributors of each individual sequence with details, visit https://doi.org/10.55876/gis8.250701bx.

## SUPPLEMENTAL MATERIAL

Supplemental figures and table spectrum.00860-26-s0001.pdf Figures S1 to S4, and Table S1.

OPEN PEER REVIEW reviewer-comments.pdf An accounting of the reviewer comments and feedback.

ASM does not own the copyrights to Supplemental Material that may be linked to, or accessed through, an article. The authors have granted ASM a non-exclusive, world-wide license to publish the Supplemental Material files. Please contact the corresponding author directly for reuse.
