# Supplementary material for: Longitudinal genomic surveillance of SARS-CoV-2 in a university microcosm reflects global evolutionary trends
Source: Microbiol Spectr. 2026 Jun 15;14(7):e00860-26. doi: 10.1128/spectrum.00860-26 (PMC13340101; doi:10.1128/spectrum.00860-26)
Supplement: Supplemental figures and table — Figures S1 to S4, and Table S1. [file spectrum.00860-26-s0001.pdf]

Table S1. Distribution of specimen type by age group and sex

	**Screening specimen (n=888)**	**Clinical specimen (n=1786)**
**Age group (Year)**		
<1-17	4 (0.45 %)	59 (3.3%)
18-30	636 (71.62 %)	1197 (67%)
31-50	37 (4.17 %)	270 (15.1%)
51-70	7 (0.79 %)	166 (9.3%)
71-97	0	91 (5.1%)
Unknown	204 (22.97%)	3 (0.2%)
**Sex**		
Female	335 (49%)	963 (54%)
Male	349 (51%)	820 (46%)

Four years of monthly detection of SARS-CoV-2 lineages in the university community

**Oct-20**	B.1.2	BA.1	BA.2.20	BA.5.6	BE.1.2	BN.1.3	XBB.1.9.2	XBB.1.42.2	**Dec-23**	**Jun-24**
B.1.2	**Apr-21**	BA.1.1	BA.2.26	BA.5.8	BF.10	BQ.1	**May-23**	XBB.1.5	BA.2.86.1	JN.1.1.6
**Nov-20**	B.1.1.7	BA.1.15	BA.2.59	BE.1	BF.5	BQ.1.1	FL.9	XBB.1.5.10	EG.5.1.1	JN.1.16
B.1	**Jul-21**	BA.1.20	BA.2.9	BE.3	**Oct-22**	BQ.1.12	XBB.1.16	XBB.1.5.59	EG.5.1.8	JN.1.65.1
B.1.1	AY.20	**Jan-22**	BA.4	BF.10	BA.4.6	BQ.1.13	XBB.1.5	XBB.1.5.72	HK.3	KP.2.3
B.1.1.222	AY.35	B	BA.4.1	BF.27	BA.4.6.1	BQ.1.6	XBB.1.5.1	XBB.1.9.1	HV.1	**Jul-24**
B.1.1.337	P.1.12	BA.1	BA.4.1.1	BF.28	BA.5.1	BU.1	XBB.1.5.13	XBB.1.9.2	JF.1	JN.1.18
B.1.1.432	**Aug-21**	BA.1.1	BA.4.4	BF.8	BA.5.1.5	CA.7	XBB.1.5.77	XBB.2.3	JN.1	JN.1.18.3
B.1.110.3	AY.100	BA.1.1.18	BA.4.6	**Aug-22**	BA.5.2.1	CK.2.1	XBB.1.9	XBB.2.4	JN.1.1	JN.1.54
B.1.139	AY.103	BA.1.15	BA.5	BA.2	BA.5.2.23	EY.1	XBB.2.3.3	XBC.1.6.1	JN.1.2	KP.1.1.1
B.1.2	AY.107	BA.1.15.2	BA.5.1	BA.2.12.1	BA.5.2.53	XBB.1	**Jun-23**	**Sep-23**	**Jan-24**	KP.2.3
B.1.240	AY.20	BA.1.20	BA.5.1.1	BA.2.75	BF.10	XBB.1.15	XBB.1.5	EG.5	BA.2.86.1	KP.3.1
B.1.396	AY.25	BA.2	BA.5.1.23	BA.2.75.2	BF.4	XBB.1.5	XBB.1.5.10	EG.5.1.1	GK.1.1	KP.3.1.1
B.1.565	AY.26	BA.2.10	BA.5.1.30	BA.4	BF.5	XBB.1.5.15	**Jul-23**	EG.5.1.3	HV.1	LB.1.7
B.1.577	AY.3	**Feb-22**	BA.5.2	BA.4.1	BF.7	XBB.1.5.17	BA.2.10.1	EG.5.1.4	JD.1.1.1	**Aug-24**
B.1.595	AY.35	BA.1	BA.5.2.1	BA.4.4	**Nov-22**	XBB.1.5.38	EG.5.1	EG.6	JN.1	JN.1
B.1.609	AY.4	BA.1.1	BA.5.3.1	BA.4.6	BA.5	XBB.1.5.51	EG.5.1.3	EG.6.1	JN.1.4	JN.1.11
**Dec-20**	AY.44	BA.1.15	BA.5.3.2	BA.5	BA.5.2	XBB.1.9	FL.1.5.1	FL.1.5.1	JN.1.45	JN.1.13.1
B.1	AY.46.4	BA.2	BA.5.5	BA.5.1	BA.5.2.23	XBB.2.12	GN.1	FU.2	JN.1.52	JN.1.15.1
B.1.110.3	AY.47	**Mar-22**	BA.5.6	BA.5.1.1	BA.5.2.34	**Feb-23**	XBB.1.16	GE.1	JN.5	JN.1.16
B.1.2	AY.54	B.1.577	BA.5.8	BA.5.1.2	BA.5.2.6	BQ.1.1	XBB.1.16.6	GJ.5	XBB.1.41.1	JN.1.16.1
B.1.234	AY.64	BA.1	BE.1	BA.5.1.23	BA.5.2.9	BQ.1.1.39	XBB.1.5	HN.1	XBC.1	JN.1.18
B.1.577	AY.75	BA.1.1	BE.3	BA.5.1.25	BE.1.1	BQ.1.1.69	XBB.2.3.8	HS.1.1	**Feb-24**	JN.1.9
B.1.596	B.1.617.2	BA.1.15	BE.5	BA.5.1.30	BF.28	BQ.1.22	XCK	HV.1	BA.2.86.1	KP.2
**Jan-21**	**Sep-21**	BA.2	BF.10	BA.5.1.6	BN.1	BQ.1.5	**Aug-23**	XBB.1.16.11	EG.5.1.3	KP.2.2
B.1	A	BA.2.3	BF.27	BA.5.10	BQ.1	XBB.1.5	EG.1.5	XBB.1.16.14	HK.3	KP.2.3
B.1.1	AY.100	BA.2.9	BF.5	BA.5.2	BQ.1.1	XBB.1.5.15	EG.5.1	XBB.1.16.15	JN.1	KP.3.1
B.1.1.222	AY.103	**Apr-22**	BF.8	BA.5.2.1	BQ.1.1.69	XBB.1.5.17	EG.5.1.1	XBB.1.16.6	JN.1.1	KP.3.1.1
B.1.1.362	AY.107	BA.1	XAF	BA.5.2.9	BQ.1.11	XBB.1.5.21	EG.5.1.3	XBB.1.5.10	JN.1.32	KP.3.5
B.1.1.487	AY.114	BA.2	XW	BA.5.3.1	BQ.1.12	XBB.1.5.33	EG.5.1.4	XBC.1.6.1	JN.1.39	LB.1
B.1.1.519	AY.118	BA.2.12.1	**Jul-22**	BA.5.5	BQ.1.13	XBB.1.5.51	EG.5.1.6	**Oct-23**	JN.1.4	LB.1.2
B.1.1.7	AY.119.2	BA.2.20	BA.2	BA.5.5.3	BQ.1.14	XBB.1.5.75	EG.5.1.9	EG.10.1	JN.1.42	LB.1.7
B.1.2	AY.122	BA.2.3	BA.2.12	BA.5.6	BQ.1.4	**Mar-23**	EG.6.1	EG.5.1	JN.1.43	LF.3.1.1
B.1.234	AY.20	BA.2.3.10	BA.2.12.1	BE.1.1	CH.1.1.25	BQ.1.1.58	FD.2	EG.5.1.3	JN.1.57	LY.1
B.1.240	AY.25	BA.2.3.4	BA.2.76	BE.1.2	XBB.2.11.1	EA.1	FL.1.5.1	HK.11	JN.1.7	MB.1.1
B.1.243	AY.25.1	BA.2.9	BA.4	BF.10	XBD	FB.1	FL.19.1	HV.1	JN.1.7.5	**Sep-24**
B.1.369	AY.26	BA.2.9.2	BA.4.1	BF.26	**Dec-22**	FL.8	FL.2	HY.1	**Mar-24**	JN.1
B.1.396	AY.3	**May-22**	BA.4.1.1	BF.5	BA.5.2	XBB.1.5	FL.21	JD.1.1	JN.1	JN.1.16
B.1.429	AY.35	BA.2	BA.4.2	BN.2	BN.1.2	XBB.1.5.11	GK.1	XBB.1.16.6	JN.1.1	JN.1.16.1
B.1.558	AY.39	BA.2.12	BA.4.4	XAS	BQ.1	XBB.1.5.15	HF.1.1	**Nov-23**	JN.1.13	JN.1.18
B.1.577	AY.39.1	BA.2.12.1	BA.4.6	**Sep-22**	BQ.1.1	XBB.1.5.17	HF.1.2	EG.10.1	JN.1.19	KP.1.1.1
B.1.595	AY.4	BA.2.18	BA.4.6.5	BA.4	BQ.1.1.1	XBB.1.5.26	HH.2	EG.5.1	JN.1.38	KP.1.1.5
B.1.596	AY.44	BA.2.23	BA.5	BA.4.6	BQ.1.11	XBB.1.5.62	HN.1	EG.5.1.6	JN.1.39	KP.2.24
R.1	AY.46.4	BA.2.3	BA.5.1	BA.5.1	BQ.1.23	**Apr-23**	HS.1	FL.1.5.1	JN.1.4	KP.3.1
**Feb-21**	AY.47	BA.2.59	BA.5.1.1	BA.5.1.2	BQ.1.3.2	B	HV.1	GK.1.1	JN.1.4.5	KP.3.1.1
B.1	AY.64	BA.2.73	BA.5.1.22	BA.5.1.23	BQ.1.8	BA.2.10.1	HZ.1	HK.3	JN.1.4.7	KS.1.1
B.1.1.7	AY.75	BA.2.9	BA.5.1.23	BA.5.1.25	CH.1.1.25	EG.4	XBB.1.16	HV.1	JN.1.9	LB.1
B.1.2	B	BA.2.9.2	BA.5.1.30	BA.5.1.30	CK.2.1.1	XBB.1	XBB.1.16.11	JD.1	**Apr-24**	LB.1.2
B.1.243	B.1.617.2	BA.4	BA.5.2	BA.5.2	ED.3	XBB.1.16	XBB.1.16.15	JD.1.1	B	LY.1
B.1.429	B.4	**Jun-22**	BA.5.2.1	BA.5.2.1	**Jan-23**	XBB.1.16.3	XBB.1.16.19	JG.3	JN.1	**Oct-24**
B.1.533	**Dec-21**	BA.2	BA.5.2.9	BA.5.2.26	BA.2	XBB.1.5	XBB.1.16.2	JN.1	JN.1.16	KP.2.2
**Mar-21**	AY.100	BA.2.12	BA.5.3.1	BA.5.2.9	BA.2.10.1	XBB.1.5.28	XBB.1.16.6	JN.9	JN.1.32	KP.2.3
B.1	AY.25.1	BA.2.12.1	BA.5.5	BA.5.5	BA.2.75.5	XBB.1.5.49	XBB.1.22	XBB.1.16	**May-24**	KP.3.1.1
B.1.1.7	B	BA.2.18	BA.5.5.3	BE.1.1	BA.5.2	XBB.1.5.62	XBB.1.41.1	XBB.1.16.15	JN.1.16	KP.3.4

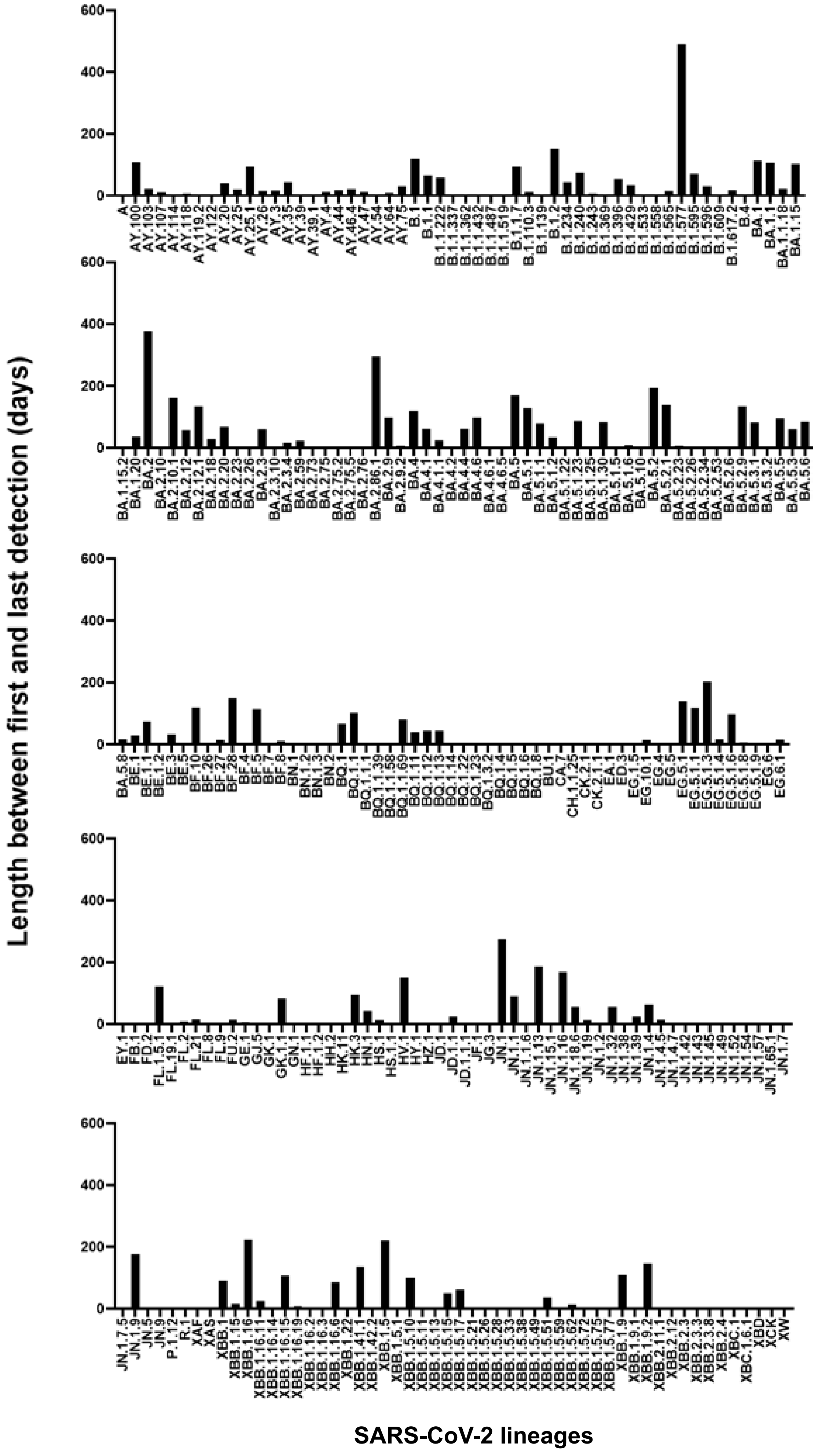
Length between first and last detection (days)
SARS-CoV-2 lineages

Sex-Based Prevalence of SARS-CoV-2 Lineages in the University Community

Female (F)

A
AY.100
AY.103
AY.114
AY.119.2
AY.122
AY.20
AY.25
AY.25.1
AY.26
AY.3
AY.35
AY.4
AY.44
AY.46.4
AY.47
AY.54
AY.75
B
B.1
B.1.1.7
B.1.110.3
B.1.2
B.1.577
B.1.609
B.1.617.2
B.4
BA.1
BA.1.1
BA.1.1.18
BA.1.15
BA.1.15.2
BA.1.20
BA.2
BA.2.10
BA.2.10.1
BA.2.12
BA.2.12.1
BA.2.18
BA.2.26
BA.2.3
BA.2.3.4
BA.2.75.2
BA.2.86.1
BA.2.9
BA.2.9.2
BA.4
BA.4.1
BA.4.1.1
BA.4.4
BA.4.6
BA.4.6.1
BA.4.6.5
BA.5
BA.5.1
BA.5.1.1
BA.5.1.2
BA.5.1.23
BA.5.1.30
BA.5.1.6
BA.5.2
BA.5.2.1
BA.5.2.23
BA.5.2.34
BA.5.2.53
BA.5.2.9
BA.5.3.1
BA.5.3.2
BA.5.5
BA.5.5.3
BA.5.6
BE.1
BE.1.1
BE.1.2
BE.3
BF.10
BF.27
BF.28
BF.5
BF.8
BN.1
BN.1.3
BN.2
BQ.1
BQ.1.1
BQ.1.1.1
BQ.1.1.58
BQ.1.1.69
BQ.1.11
BQ.1.12
BQ.1.22
BQ.1.23
BQ.1.4
BQ.1.8
CH.1.1.25
CK.2.1.1
ED.3
EG.1.5
EG.10.1
EG.4
EG.5
EG.5.1
EG.5.1.1
EG.5.1.3
EG.5.1.4
EG.5.1.6
EG.5.1.9
EG.6
EG.6.1
EY.1
FL.1.5.1
FL.2
FL.21
FL.9
FU.2
GE.1
GK.1.1
HK.3
HN.1
HS.1
HS.1.1
HV.1
HZ.1
JD.1.1
JD.1.1.1
JN.1
JN.1.1
JN.1.11
JN.1.13.1
JN.1.15.1
JN.1.16
JN.1.16.1
JN.1.18
JN.1.32
JN.1.38
JN.1.39
JN.1.4
JN.1.4.5
JN.1.42
JN.1.43
JN.1.57
JN.1.7
JN.1.7.5
JN.1.9
JN.5
JN.9
KP.1.1.1
KP.1.1.5
KP.2
KP.2.24
KP.2.3
KP.3.1
KP.3.1.1
KP.3.3
KP.3.5
LB.1
LB.1.2
LB.1.7
LY.1
MB.1.1
XAF
XAS
XBB.1
XBB.1.15
XBB.1.16
XBB.1.16.11
XBB.1.16.14
XBB.1.16.15
XBB.1.16.2
XBB.1.16.6
XBB.1.22
XBB.1.41.1
XBB.1.42.2
XBB.1.5
XBB.1.5.1
XBB.1.5.10
XBB.1.5.11
XBB.1.5.13
XBB.1.5.15
XBB.1.5.17
XBB.1.5.26
XBB.1.5.49
XBB.1.5.51
XBB.1.5.59
XBB.1.5.62
XBB.1.5.77
XBB.1.9
XBB.1.9.1
XBB.1.9.2
XBB.2.3
XBB.2.3.3
XBB.2.4
XBC.1.6.1
XBD
XW

F-specific

A
AY.122
AY.54
B.4
BA.1.15.2
BA.2.10
BA.2.3.4
BA.2.75.2
BA.2.9.2
BA.4.6.5
BA.5.2.34
BA.5.2.53
BA.5.3.2
BN.1
BN.1.3
BQ.1.1.58
BQ.1.22
BQ.1.23
BQ.1.4
BQ.1.8
CK.2.1.1
ED.3
EG.1.5
EG.10.1
EG.4
EG.5
EG.5.1.6
EG.5.1.9
EG.6
EY.1
HN.1
HS.1.1
HZ.1
JD.1.1
JD.1.1.1
JN.1.11
JN.1.13.1
JN.1.15.1
JN.1.32
JN.1.38
JN.1.39
JN.1.42
JN.1.43
JN.1.57
JN.1.7
JN.1.7.5
JN.1.9
JN.5
JN.9
KP.1.1.5
KP.2
KP.3.3
KP.3.5
MB.1.1
XAF
XAS
XBB.1
XBB.1.16.14
XBB.1.16.2
XBB.1.22
XBB.1.42.2
XBB.1.5.1
XBB.1.5.11
XBB.1.5.13
XBB.1.5.26
XBB.1.5.49
XBB.1.5.59
XBB.1.5.62
XBB.1.5.77
XBB.1.9
XBB.2.3
XBB.2.3.3
XBB.2.4
XBD

Male (M)

AY.100
AY.103
AY.107
AY.114
AY.118
AY.119.2
AY.20
AY.25
AY.25.1
AY.26
AY.3
AY.35
AY.39
AY.39.1
AY.4
AY.44
AY.46.4
AY.47
AY.64
AY.75
B
B.1
B.1.1.7
B.1.110.3
B.1.2
B.1.234
B.1.240
B.1.596
B.1.609
B.1.617.2
BA.1
BA.1.1
BA.1.1.18
BA.1.15
BA.1.20
BA.2
BA.2.10.1
BA.2.12
BA.2.12.1
BA.2.18
BA.2.20
BA.2.23
BA.2.26
BA.2.3
BA.2.3.10
BA.2.59
BA.2.73
BA.2.75
BA.2.75.5
BA.2.76
BA.2.86.1
BA.2.9
BA.4
BA.4.1
BA.4.1.1
BA.4.2
BA.4.4
BA.4.6
BA.4.6.1
BA.5
BA.5.1
BA.5.1.1
BA.5.1.2
BA.5.1.22
BA.5.1.23
BA.5.1.25
BA.5.1.30
BA.5.1.5
BA.5.1.6
BA.5.10
BA.5.2
BA.5.2.1
BA.5.2.23
BA.5.2.26
BA.5.2.6
BA.5.2.9
BA.5.3.1
BA.5.5
BA.5.5.3
BA.5.6
BA.5.8
BE.1
BE.1.1
BE.1.2
BE.3
BE.5
BF.10
BF.26
BF.27
BF.28
BF.4
BF.5
BF.7
BF.8
BN.1.2
BN.2
BQ.1
BQ.1.1
BQ.1.1.1
BQ.1.1.39
BQ.1.1.69
BQ.1.11
BQ.1.12
BQ.1.13
BQ.1.14
BQ.1.3.2
BQ.1.5
BQ.1.6
BU.1
CA.7
CH.1.1.25
CK.2.1
EA.1
EG.5.1
EG.5.1.1
EG.5.1.3
EG.5.1.4
EG.5.1.8
EG.6.1
FB.1
FD.2
FL.1.5.1
FL.19.1
FL.2
FL.21
FL.8
FL.9
FU.2
GE.1
GJ.5
GK.1
GK.1.1
GN.1
HF.1.1
HF.1.2
HH.2
HK.11
HK.3
HS.1
HV.1
HY.1
JD.1
JF.1
JG.3
JN.1
JN.1.1
JN.1.1.6
JN.1.13
JN.1.16
JN.1.16.1
JN.1.18
JN.1.18.3
JN.1.19
JN.1.2
JN.1.4
JN.1.4.5
JN.1.4.7
JN.1.45
JN.1.52
JN.1.65.1
KP.1.1.1
KP.2.2
KP.2.24
KP.2.3
KP.3.1
KP.3.1.1
KP.3.4
KS.1.1
LB.1
LB.1.2
LB.1.7
LF.3.1.1
LY.1
P.1.12
XBB.1.15
XBB.1.16
XBB.1.16.11
XBB.1.16.15
XBB.1.16.19
XBB.1.16.3
XBB.1.16.6
XBB.1.41.1
XBB.1.5
XBB.1.5.10
XBB.1.5.15
XBB.1.5.17
XBB.1.5.21
XBB.1.5.28
XBB.1.5.33
XBB.1.5.38
XBB.1.5.51
XBB.1.5.72
XBB.1.5.75
XBB.1.9.1
XBB.1.9.2
XBB.2.11.1
XBB.2.12
XBB.2.3.8
XBC.1
XBC.1.6.1
XCK
XEC
XEF
XW

M-specific

AY.107
AY.118
AY.39
AY.39.1
AY.64
BA.2.20
BA.2.23
BA.2.3.10
BA.2.59
BA.2.73
BA.2.75
BA.2.75.5
BA.2.76
BA.4.2
BA.5.1.22
BA.5.1.25
BA.5.1.5
BA.5.10
BA.5.2.26
BA.5.2.6
BA.5.8
BE.5
BF.26
BF.4
BF.7
BN.1.2
BQ.1.1.39
BQ.1.13
BQ.1.14
BQ.1.3.2
BQ.1.5
BQ.1.6
BU.1
CA.7
CK.2.1
EA.1
EG.5.1.8
FB.1
FD.2
FL.19.1
FL.8
GJ.5
GK.1
GN.1
HF.1.1
HF.1.2
HH.2
HK.11
HY.1
JD.1
JF.1
JG.3
JN.1.1.6
JN.1.13
JN.1.18.3
JN.1.19
JN.1.2
JN.1.4.7
JN.1.45
JN.1.52
JN.1.65.1
KP.2.2
KP.3.4
KS.1.1
LF.3.1.1
P.1.12
XBB.1.16.19
XBB.1.16.3
XBB.1.5.21
XBB.1.5.28
XBB.1.5.33
XBB.1.5.38
XBB.1.5.72
XBB.1.5.75
XBB.2.11.1
XBB.2.12
XBB.2.3.8
XBC.1

Unknown (U)

B.1
B.1.1
B.1.1.222
B.1.1.337
B.1.1.362
B.1.1.432
B.1.1.487
B.1.1.519
B.1.1.7
B.1.110.3
B.1.139
B.1.2
B.1.234
B.1.240
B.1.243
B.1.369
B.1.396
B.1.429
B.1.533
B.1.558
B.1.565
B.1.577
B.1.595
B.1.596
BA.4.1.1
BA.5.2
BA.5.2.1
R.1

U-specific

B.1.1
B.1.1.222
B.1.1.337
B.1.1.362
B.1.1.432
B.1.1.487
B.1.1.519
B.1.139
B.1.243
B.1.369
B.1.396
B.1.429
B.1.533
B.1.558
B.1.565
B.1.595
R.1

Figure S4

Age-based distribution of SARS-CoV-2 Lineages in the University community

<1Y: BA.1.1, BA.2.12.1, BA.4.1, BA.5.3.1, BA.5.5, BF.8

1Y: BA.2.12.1, BA.4.2, BA.5.3.1, BA.5.5, BF.10

2Y: BA.2, BA.5.5, BA.5.6

3Y: BA.2, BA.2.12.1

4Y: BA.1.1, BA.2.12.1

5Y: BA.2, BA.2.12.1, BA.4

6Y: BA.5.2.1

7Y: BA.2.12.1, BA.5.5

8Y: BA.1.1

9Y: BA.1.1, BA.2.12.1, BA.4.1

10Y: BA.2, BA.2.12.1, BA.5.5

11Y: BA.2.12.1

12Y: BA.2.12.1, BA.4, BA.4.1, BF.28

13Y: BA.2.12, BA.4.4, BA.5.1.1

14Y: BA.1.15, BA.2, BA.2.12.1, BA.4

16Y: BA.1.15, BA.2.12.1

17Y: AY.26, BA.2.12.1, BA.4, BA.4.1.1, BA.5.2.1, BA.5.5, EG.5.1.8, KP.2.3, P.1.12, XBB.1.5

18Y: A, AY.100, AY.103, AY.20, AY.25, AY.3, AY.35, AY.4, AY.44, AY.46.4, AY.47, AY.64, AY.75, B.1.1.7, B.1.2, B.1.617.2, BA.1, BA.1.1, BA.1.15, BA.2.10.1, BA.2.12.1, BA.2.75, BA.2.86.1, BA.4, BA.4.1, BA.4.6, BA.5.1, BA.5.1.1, BA.5.1.2, BA.5.1.23, BA.5.2, BA.5.2.1, BA.5.2.34, BA.5.5, BA.5.6, BE.1.2, BF.10, BF.27, BF.5, BN.2, BQ.1, BQ.1.1, CA.7, CH.1.1.25, EA.1, EG.5, EG.5.1, EG.5.1.1, EG.5.1.4, EG.5.1.8, EG.6.1, FL.1.5.1, GK.1.1, HN.1, HV.1, JN.1, JN.1.16, JN.1.16.1, JN.1.18, JN.1.4, JN.9, KP.2.3, KP.3.1, KP.3.1.1, LB.1.2, XAS, XBB.1, XBB.1.16, XBB.1.16.1, XBB.1.16.6, XBB.1.41.1, XBB.1.5, XBB.1.5.10, XBB.1.5.15, XBB.1.5.17, XBB.1.5.33, XBB.1.5.51

19Y: AY.100, AY.103, AY.114, AY.118, AY.122, AY.20, AY.25, AY.26, AY.3, AY.4, AY.44, AY.46.4, AY.47, B.1.1.7, B.1.2, B.1.617.2, BA.1, BA.1.1, BA.1.15, BA.1.20, BA.2, BA.2.10.1, BA.2.12.1, BA.2.3.4, BA.2.86.1, BA.2.9, BA.4, BA.4.1.1, BA.4.6, BA.4.6.1, BA.5.1, BA.5.1.23, BA.5.1.25, BA.5.1.30, BA.5.2.1, BA.5.2.9, BA.5.3.1, BA.5.5, BA.5.6, BE.1.1, BF.5, BQ.1, BQ.1.1, BQ.1.1.69, BQ.1.14, BQ.1.22, BU.1, EG.1.5, EG.5.1.1, EG.5.1.3, EG.5.1.4, FL.1.5.1, FL.2, FL.21, FL.8, FU.2, HF.1.2, HV.1, JD.1.1, JN.1, JN.1.1, JN.1.13.1, JN.1.16, JN.1.16.1, JN.1.18, JN.1.19, JN.1.39, JN.1.4, JN.1.4.5, JN.1.42, JN.1.57, JN.1.7.5, JN.1.9, KP.1.1.1, KP.2.3, KP.3.1, KP.3.1.1, LB.1, LB.1.2, LY.1, XBB.1, XBB.1.16.1, XBB.1.16.1, XBB.1.16.1, XBB.1.16.6, XBB.1.5, XBB.1.5.10, XBB.1.5.11, XBB.1.5.17, XBB.1.5.28, XBB.1.5.38, XBB.1.5.51, XBB.1.9.1, XBB.2.3

20Y: AY.100, AY.103, AY.118, AY.20, AY.25, AY.25.1, AY.26, AY.3, AY.35, AY.39.1, AY.4, AY.44, AY.46.4, AY.47, AY.75, B, B.1, B.1.1.7, B.1.2, B.1.617.2, B.4, BA.1, BA.1.1, BA.1.1.18, BA.1.15, BA.1.15.2, BA.2, BA.2.10.1, BA.2.12.1, BA.2.75.2, BA.2.86.1, BA.2.9, BA.4, BA.4.1.1, BA.4.6, BA.5.1, BA.5.2.1, BA.5.2.53, BA.5.3.1, BA.5.5, BA.5.5.3, BE.1, BE.1.1, BE.5, BF.27, BF.5, BN.1.3, BQ.1, BQ.1.1, BQ.1.1.39, BQ.1.11, BQ.1.12, BQ.1.4, ED.3, EG.4, EG.5.1, EG.5.1.1, EG.5.1.3, EG.5.1.4, FD.2, FL.1.5.1, FL.19.1, HH.2, HS.1, HV.1, HY.1, HZ.1, JD.1, JN.1, JN.1.1, JN.1.16, JN.1.18, JN.1.32, JN.1.38, JN.1.4, JN.1.4.5, JN.1.43, JN.1.52, KP.3.1, KP.3.1.1, KP.3.4, LB.1.2, LB.1.7, XBB.1, XBB.1.15, XBB.1.16, XBB.1.16.1, XBB.1.16.1, XBB.1.16.6, XBB.1.5, XBB.1.5.15, XBB.1.5.17, XBB.1.5.26, XBB.1.5.49, XBB.1.5.51, XBB.1.5.59, XBB.2.4, XEF

21Y: AY.100, AY.103, AY.107, AY.114, AY.119.2, AY.20, AY.25, AY.25.1, AY.26, AY.3, AY.35, AY.39, AY.4, AY.44, AY.46.4, AY.47, AY.75, B.1, B.1.1.7, B.1.2, BA.1, BA.1.1, BA.1.1.18, BA.1.15, BA.2, BA.2.12.1, BA.2.3.4, BA.2.86.1, BA.4.1, BA.4.4, BA.4.6, BA.4.6.1, BA.5.1, BA.5.1.1, BA.5.1.2, BA.5.1.23, BA.5.1.6, BA.5.2, BA.5.2.1, BA.5.5, BA.5.6, BE.1.2, BF.10, BF.5, BN.1, BN.2, BQ.1, BQ.1.1, BQ.1.13, BQ.1.23, EG.5.1.1, EG.5.1.3, EG.5.1.4, EG.5.1.6, EG.6, FL.1.5.1, GJ.5, HK.3, HV.1, JN.1, JN.1.16, JN.1.18, JN.1.4, KP.2.24, KP.3.1, KP.3.1.1, LB.1, LB.1.2, LF.3.1.1, XBB.1, XBB.1.16, XBB.1.16.6, XBB.1.41.1, XBB.1.5, XBB.1.5.1, XBB.1.5.17, XBB.1.5.21, XBB.1.5.51, XBB.1.9.1, XBB.1.9.2, XBB.2.11.1

22Y: AY.100, AY.103, AY.119.2, AY.20, AY.25, AY.26, AY.4, AY.44, AY.46.4, AY.64, AY.75, B.1, B.1.1.7, B.1.2, B.1.234, B.1.609, B.1.617.2, BA.1, BA.1.1, BA.1.1.18, BA.1.15, BA.2, BA.2.12.1, BA.2.3, BA.2.73, BA.2.75.5, BA.2.86.1, BA.4, BA.4.1, BA.5, BA.5.1, BA.5.1.1, BA.5.1.22, BA.5.1.23, BA.5.1.30, BA.5.2, BA.5.2.1, BA.5.5, BA.5.5.3, BA.5.6, BE.3, BF.27, BQ.1, BQ.1.1, EG.10.1, EG.5.1.3, FL.1.5.1, FL.2, HS.1, HV.1, JN.1, JN.1.1, JN.1.15.1, JN.1.16, JN.1.16.1, JN.1.19, JN.1.4, JN.1.4.7, JN.1.45, KP.2.3, KP.3.1, KP.3.1.1, KP.3.3, LB.1.2, XBB.1, XBB.1.16, XBB.1.16.1, XBB.1.5, XBB.1.5.13, XBB.1.5.17, XBB.1.5.51, XBB.1.5.62, XBB.1.5.77, XBB.1.9, XBB.2.3.3, XBC.1

23Y: AY.100, AY.25, AY.26, AY.3, AY.35, AY.44, AY.46.4, B, B.1, B.1.1.7, B.1.110.3, B.1.2, B.1.609, B.1.617.2, BA.1.1, BA.1.15, BA.2, BA.2.12.1, BA.2.3.10, BA.2.86.1, BA.4.1, BA.4.1.1, BA.4.4, BA.4.6, BA.5.1, BA.5.1.1, BA.5.1.2, BA.5.1.5, BA.5.2, BA.5.2.1, BA.5.2.26, BA.5.3.1, BA.5.5, BA.5.6, BF.10, BQ.1.1, BQ.1.1.69, BQ.1.23, CH.1.1.25, CK.2.1, EG.5.1.1, EG.5.1.3, EG.5.1.6, FL.1.5.1, FL.9, FU.2, GE.1, HS.1.1, HV.1, JN.1, JN.1.7, JN.1.9, KP.1.1.1, KP.3.1, KP.3.1.1, MB.1.1, XBB.1.5, XBB.1.5.17, XBB.1.5.51

24Y: AY.100, AY.25, B, B.1.1.7, BA.1.1, BA.1.1.18, BA.1.15, BA.2, BA.2.10, BA.2.12.1, BA.2.3, BA.2.86.1, BA.4.1, BA.5.1, BA.5.1.1, BA.5.1.2, BA.5.1.23, BA.5.1.6, BA.5.10, BA.5.2, BA.5.2.1, BA.5.3.2, BE.1.2, BF.5, BQ.1, BQ.1.1, CK.2.1.1, EG.5.1.3, EG.5.1.8, FB.1, HK.11, HV.1, JN.1, KP.1.1.1, KP.2.3, KP.3.1, KP.3.1.1, XBB.1, XBB.1.15, XBB.1.16.2, XBB.1.5, XBB.1.5.10, XBB.1.5.17, XBB.1.5.51, XBB.1.5.72, XBB.1.5.75, XBD

25Y: AY.103, AY.25, AY.44, AY.54, B.1.2, BA.1, BA.1.1, BA.1.15, BA.1.20, BA.2, BA.2.12.1, BA.2.86.1, BA.4, BA.5, BA.5.1, BA.5.1.30, BA.5.2.1, BA.5.5.3, BA.5.6, BE.1, BF.26, BF.28, BF.4, BQ.1.1, EG.6.1, FL.21, FL.9, JN.1, JN.1.1, JN.1.32, KP.2.24, KP.2.3, KP.3.5, XBB.1.22, XBB.1.5, XBB.1.5.17, XBB.1.5.51

26Y: AY.25.1, AY.75, B.1.1.7, B.1.240, BA.1.1, BA.2, BA.2.12.1, BA.2.86.1, BA.4, BA.5.1, BA.5.1.1, BA.5.1.23, BA.5.2.9, BA.5.5.3, BA.5.6, BQ.1, BQ.1.1, BQ.1.1.39, EG.5.1.3, FL.1.5.1, FL.9, GK.1, JN.1, JN.1.16, JN.1.65.1, KP.1.1.5, LB.1.2, LB.1.7, XBB.1.16.6, XBB.1.5, XBB.1.5.51

27Y: AY.25, B.1.577, BA.1, BA.1.1, BA.1.15, BA.2, BA.2.12.1, BA.4, BA.4.1, BA.4.1.1, BA.5, BA.5.1, BA.5.1.25, BA.5.2.1, BA.5.2.23, BA.5.3.1, BA.5.5, BA.5.5.3, BA.5.6, BQ.1, BQ.1.1, BQ.1.6, EG.5.1, EG.5.1.3, EG.6.1, JG.3, JN.1, JN.1.11, JN.1.16, JN.1.18.3, JN.1.4, KP.2.3, KP.3.1.1, LB.1.7, LY.1, XBB.1.15, XBB.1.16.6, XBB.1.5, XBB.2.12, XBB.2.3.8, XW

28Y: AY.103, AY.3, B.1, B.1.234, BA.1.1, BA.1.15, BA.2, BA.2.12.1, BA.2.86.1, BA.4.6, BA.5.1, BA.5.1.1, BA.5.2, BA.5.2.23, BA.5.6, BE.3, BF.26, BF.7, BQ.1.1.1, HV.1, JN.1, JN.1.32, KP.2.2, KP.2.3, KP.3.1, KS.1.1, XBB.1.16.1, XBB.1.16.6, XBB.1.5, XBB.1.5.10, XBB.1.5.15, XBB.1.5.51, XBB.1.5.75, XCK, XEC

29Y: B.1.1.7, BA.1.1, BA.1.15, BA.2, BA.2.12.1, BA.2.76, BA.4, BA.4.6, BA.5.1, BA.5.2.1, BA.5.5, BA.5.6, BQ.1.3.2, EG.5.1.9, FL.1.5.1, FL.9, GE.1, HK.3, HN.1, HV.1, JN.1, JN.1.18, KP.2, KP.2.3, KP.3.1, XBB.1.15, XBB.1.16, XBB.1.5.51, XBB.1.9, XBB.1.9.2, XW

30Y: AY.103, AY.25, B, BA.1, BA.1.1, BA.1.15, BA.1.20, BA.2, BA.2.12.1, BA.2.26, BA.2.3, BA.4.1, BA.5.2, BA.5.2.9, BA.5.3.1, BA.5.6, BE.3, BF.10, BF.5, EG.5.1, GN.1, JD.1.1, JN.1, JN.1.7.5, KP.2.3, KP.3.1, XBB.1.16, XBB.1.5, XBB.1.5.51

31Y: AY.44, BA.1.1, BA.1.15, BA.2.12.1, BA.2.26, BA.5, BA.5.1.1, BA.5.6, BE.1, BQ.1.1.1, BQ.1.13, BQ.1.5, EG.10.1, EG.5.1.3, HF.1.1, JN.1, XBB.1.5, XBB.1.5.51, XBB.1.5.62

32Y: AY.100, BA.2, BA.2.12.1, BA.2.59, BA.2.86.1, BA.2.9, BA.4, BA.4.1, BA.5.1, BA.5.2.1, BA.5.2.6, BA.5.3.1, BA.5.6, BE.1, JN.1, JN.1.9

33Y: AY.100, AY.25, AY.3, BA.2, BA.2.12.1, BA.2.9, BA.5.1.1, BA.5.2.1, BA.5.5, BN.1.2, BQ.1.12, EG.5.1, FL.1.5.1, JF.1, JN.1.2, JN.1.39, KP.2.2, XBB.1.16.1, XBC.1.6.1

34Y: BA.1, BA.1.1, BA.2, BA.2.12.1, BA.5.1, BA.5.1.1, BA.5.1.30, BA.5.2, BA.5.6, BF.10, EY.1, JN.1, JN.5, KP.3.1.1, XBB.1.5

35Y: BA.1.1, BA.2, BA.2.12.1, BA.4.1.1, BA.5.2, BF.10, JN.1.1.6, JN.1.13, JN.1.16, KP.2.3, XBB.1.9.2

36Y: AY.75, BA.1, BA.1.15, BA.2, BA.2.12.1, BA.2.18, BA.4.1.1, BA.5.1, BA.5.2, BA.5.2.1, KP.2.3, XBB.1.16.3

37Y: B.1.2, BA.1, BA.1.1, BA.2, BA.2.9.2, BA.4, BA.4.1, BA.5.1.30, BA.5.2.1, BA.5.5, BA.5.6, BF.10, BQ.1.1, EG.5.1.1, XBB.1.42.2

38Y: B.1.596, BA.1.15, BA.2.12.1, BA.2.18, BA.4.1, BA.5, BA.5.1, BA.5.1.1, JD.1.1.1, XBB.1.5, XBC.1.6.1

39Y: BA.1.1, BA.2, BA.2.12, BA.2.12.1, BA.2.59, BA.2.9, BA.5.5, BF.10, JN.1

40Y: BA.1.1, BA.2, BA.2.12.1, BA.4.1, BA.5.1.1, BA.5.5, XBB.1.16.6

41Y: BA.1.1, BA.1.15, BA.2, BA.2.12, BA.2.12.1, BA.5.1, BA.5.1.1, BA.5.6, BF.8

42Y: B.1.234, BA.1.1, BA.1.15, BA.2.12.1, BA.4.1, BA.5.1, BA.5.2.1, BE.3, XAF

43Y: AY.4, BA.1, BA.1.1, BA.2, BA.2.12.1, BA.2.9, BA.2.9.2, BA.4.1, BA.5.1.1, BA.5.2, BA.5.5

44Y: BA.1.15, BA.5.1.1, BQ.1.1

45Y: AY.25, BA.1.15, BA.2.12.1, BA.5.1, BA.5.1.1, BA.5.1.30, BA.5.2.1, BA.5.5, BF.10

46Y: BA.2, BA.2.12, BA.2.12.1, BA.4.1, BA.4.1.1, BA.5, BA.5.1.1, BA.5.3.1, BQ.1.1.58, KP.2.3

47Y: AY.100, AY.25, BA.1, BA.1.1, BA.2.12.1, BA.5.1, BA.5.1.1, BA.5.1.30, BA.5.5, BA.5.6

48Y: BA.1.1, BA.1.15, BA.2.12.1, BA.4, BA.4.1, BA.5.2.1, BA.5.2.9

49Y: AY.47, BA.1.15, BA.2, BA.2.12.1, BA.4.1, BQ.1.1

50Y: AY.26, AY.44, BA.1, BA.1.1, BA.1.15, BA.2, BA.2.12.1, BA.4.1, BA.5.1.30, BA.5.6, BA.5.8, BE.1

51Y: BA.1.1, BA.2, BA.2.12.1, BA.5, BA.5.2.1, BA.5.5, BF.10

52Y: BA.2, BA.2.12.1, BA.5, BA.5.1, BA.5.1.1, BA.5.1.30, JN.1

53Y: BA.1.1, BA.2, BA.2.12.1, BA.4.1, BA.5.1, XBB.1.5

54Y: BA.2, BA.2.12.1, BA.2.9, BA.4.6, BE.1

55Y: AY.75, BA.2.12.1, BA.5.5

56Y: BA.2, BA.2.12.1, BA.5.5

57Y: BA.2, BA.2.12.1, BA.4, BA.5.1, BA.5.1.1, BQ.1

58Y: AY.100, B.1, BA.1.1, BA.2.12.1, BA.2.20, BA.4.1, BA.5.1.1, BA.5.8

59Y: BA.1.1, BA.1.15, BA.2, BA.2.12.1, XBB.1.5

60Y: BA.2.12.1, BA.2.3, BA.4, BA.5, BA.5.2.1, BE.1

61Y: BA.2, BA.2.12.1, BA.2.9, BA.4, BA.5.2.1, BE.1, BQ.1.8

62Y: BA.2.12.1, BA.5.1.1, BA.5.2.1, BA.5.6

63Y: BA.2.12.1, BA.4, BA.5.1.1

64Y: BA.2, BA.2.12.1, BA.4.1, BA.5.1.1, BA.5.5, BA.5.6

65Y: AY.25, BA.2, BA.2.12, BA.2.12.1, BA.4, BA.4.1, BA.5.1.1, BA.5.6

66Y: BA.2, BA.2.12.1, BA.5.1, BA.5.2.1, BA.5.5, BA.5.6

67Y: BA.1.15, BA.2, BA.2.12.1, BA.2.23, BA.5.1.1

68Y: BA.1.1, BA.2.12.1, BA.2.18, BA.2.20, BA.5.1.23, BA.5.5

69Y: BA.2, BA.5.1, BA.5.2, BA.5.2.1, BA.5.5

70Y: BA.2, BA.2.12, BA.2.12.1, BA.2.18, BA.5.1, BA.5.5

71Y: BA.1.15, BA.2.12.1, BA.2.9.2, BA.4.4

72Y: BA.1.1, BA.2, BA.2.12.1, BA.2.3, BA.4.6.5, BA.5, BA.5.2.1, BA.5.5

73Y: BA.1.15, BA.2.12.1, BA.2.9.2, BA.4, BA.5.1.1, BA.5.2.1

74Y: BA.2, BA.2.12.1, BA.5.1.30, BA.5.5

75Y: BA.1.1, BA.2, BA.2.12.1, BA.4.6, BA.5.1.1, BA.5.3.1, BA.5.5

76Y: BA.1.15, BA.2, BA.5.1.1, BA.5.2.1

77Y: BA.1.15, BA.4, BA.5.5, BF.8

78Y: BA.5.5, BE.3

79Y: BA.5, BA.5.2, BA.5.6, BF.10

80Y: BA.2, BA.4, BA.4.1, BA.5.6, BE.3

81Y: BA.1.1, BA.5.1.1

82Y: BA.2, BA.4.4, BA.5.2.1

83Y: B.1.577, BA.1.15, BA.5.1

84Y: BA.1, BA.2.12.1

85Y: BA.1.1, BA.2, BA.2.20, BA.4.1, BA.5.2.1, BE.1, BF.10

86Y: BA.2.9

87Y: BA.2, BA.2.9

88Y: BA.4.1

89Y: BA.5.5

91Y: BA.2.12.1, BA.5.1.1

92Y: BA.2

94Y: BA.1.1, BA.2

95Y: BE.3

97Y: BA.2.12.1

Unknown: B.1, B.1.1, B.1.1.222, B.1.1.337, B.1.1.362, B.1.1.432, B.1.1.487, B.1.1.519, B.1.1.7, B.1.110.3, B.1.139, B.1.2, B.1.234, B.1.240, B.1.243, B.1.369, B.1.396, B.1.429, B.1.533, B.1.558, B.1.565, B.1.577, B.1.595, B.1.596, BA.4.1.1, BA.5.2, BA.5.2.1, R.1

Supplemental figure legends

Figure S1. Monthly distribution of the cumulative 2,674 SARS-CoV-2 lineages identified in the university community over four years.

Figure S2. Detection span of SARS-CoV-2 lineages in the university community. Detection span refers to the number of days between the first and last detection of each lineage during four years of genomic surveillance in the community.

Figure S3. Sex-based distribution and identity of 296 distinct SARS-CoV-2 lineages that persisted in the university community over a four-year period.

Figure S4. Age-based distribution and identity of SARS-CoV-2 lineages that persisted in the university community over a four-year period.
