# Supplementary material for: Longitudinal genomic surveillance of SARS-CoV-2 in a university microcosm reflects global evolutionary trends
Source: Microbiol Spectr. 2026 Jun 15;14(7):e00860-26. doi: 10.1128/spectrum.00860-26 (PMC13340101; doi:10.1128/spectrum.00860-26)
Supplement: Reviewer comments [file reviewer-comments.pdf]

AMERICAN SOCIETY FOR MICROBIOLOGY | Microbiology Spectrum

Longitudinal genomic surveillance of SARS-CoV-2 in a university microcosm reflects global evolutionary trends

Sankar Chaki, Melissa Kahl-McDonagh, Ben Newman, Loni Taylor, Rebecca Fischer, Blake Hanson, Marlisa Hardy, Jo Culpepper, Eric Boerwinkle, and Kurt Zuelke

Corresponding Author(s): Sankar Chaki, Texas A&M University

Review Timeline:

Submission Date:	March 10, 2026
Editorial Decision:	March 19, 2026
Revision Received:	March 20, 2026
Accepted:	March 23, 2026

Editor: JJ Miranda

Reviewer(s): The reviewers have opted to remain anonymous.

Transaction Report:

(Note: With the exception of the correction of typographical or spelling errors that could be a source of ambiguity, letters and reports are not edited. The original formatting of letters and referee reports may not be reflected in this compilation.)

DOI: https://doi.org/10.1128/spectrum.00860-26

Re: Spectrum00860-26 (Longitudinal genomic surveillance of SARS-CoV-2 in a university microcosm reflects global evolutionary trends)

Dear Dr. Sankar Prasad Chaki:

Thank you for the privilege of reviewing your work. Below you will find my comments, instructions from the Spectrum editorial office, and the reviewer comments.

Thank you for transferring your submission detailing the longitudinal surveillance of SARS-CoV-2 variants in a university. The manuscript, prior reviews, and responses have been examined by a Senior Editor. The revised manuscript addresses prior critiques involving rigor and reproducibility. The large longitudinal data reveals findings that contribute to the field of SARS-CoV-2 genomics.

I am pleased to inform you that your manuscript has been editorially accepted for publication. However, there are a few additional questions in the submission form that need to be answered before the final decision. Once these are completed, please return your submission so that I can move your paper forward to acceptance.

Please return the manuscript within 60 days; if you cannot complete the modification within this time period, please contact me. If you do not wish to modify the manuscript and prefer to submit it to another journal, notify me immediately so that the manuscript may be formally withdrawn from consideration by Spectrum.

Revision Guidelines
To submit your modified manuscript, log into the submission site at https://spectrum.msubmit.net/cgi-bin/main.plex. Go to Author Tasks and click the appropriate manuscript title to begin. The information you entered when you first submitted the paper will be displayed; update this as necessary. Note the following requirements:

• Upload point-by-point responses to the issues raised by the reviewers in a file named "Response to Reviewers," NOT in your cover letter.
• Upload a compare copy of the manuscript (without figures) as a "Marked-Up Manuscript" file.
• Upload a clean .DOC/.DOCX version of the revised manuscript and remove the previous version.
• Each figure must be uploaded as a separate, editable, high-resolution file (TIFF or EPS preferred), and any multipanel figures must be assembled into one file.
• Any supplemental material intended for posting by ASM should be uploaded with their legends separate from the main manuscript. You can combine all supplemental material into one file (preferred) or split it into a maximum of 10 files with all associated legends included.

For complete guidelines on revision requirements, see our Submission and Review Process webpage. Submission of a paper that does not conform to guidelines may delay acceptance of your manuscript.

Data availability: ASM policy requires that data be available to the public upon online posting of the article, so please verify all links to sequence records, if present, and make sure that each number retrieves the full record of the data. If a new accession number is not linked or a link is broken, provide Spectrum production staff with the correct URL for the record. If the accession numbers for new data are not publicly accessible before the expected online posting of the article, publication may be delayed; please contact production staff (Spectrum@asmusa.org) immediately with the expected release date.

Publication Fees: For information on publication fees and which article types are subject to charges, visit our website. If your manuscript is accepted for publication and any fees apply, you will be contacted separately about payment during the production process; please follow the instructions in that e-mail. Arrangements for payment must be made before your article is published.

ASM Membership: Corresponding authors may join or renew ASM membership to obtain discounts on publication fees. Need to upgrade your membership level? Please contact Customer Service at Service@asmusa.org.

The ASM Journals program strives for constant improvement in our submission and publication process. Please tell us how we can improve your experience by taking this quick Author Survey.

Thank you for submitting your paper to Spectrum.

Sincerely,
JJ Miranda
Editor
Microbiology Spectrum

**From:**	em.onehlt.0.97654b.7c0f3414@editorialmanager.com on behalf of One Health
**To:**	Chaki, Sankar P
**Subject:**	Decision on submission to One Health
**Date:**	Wednesday, November 12, 2025 1:02:56 PM
**Attachments:**	Comments.docx

This Message Is From an External Sender
This message came from outside your organization.

CC: p.rampelotto@elsevier.com

Manuscript Number: ONEHLT-D-25-01096
Microcosm of a pandemic: what four years of genomic surveillance in a university community reveal about SARS-CoV-2

Dear Dr. Chaki,

Thank you for submitting your manuscript to One Health.

I have completed my evaluation of your manuscript. The reviewers recommend reconsideration of your manuscript following major revision and modification. I invite you to resubmit your manuscript after addressing the comments below. Please resubmit your revised manuscript by Dec 03, 2025.

When revising your manuscript, please consider all issues mentioned in the reviewers' comments carefully: please outline every change made in response to their comments and provide suitable rebuttals for any comments not addressed. Please note that your revised submission may need to be re-reviewed.

To submit your revised manuscript, please log in as an author at https://www.editorialmanager.com/onehlt/, and navigate to the "Submissions Needing Revision" folder under the Author Main Menu.

Research Elements (optional)
This journal encourages you to share research objects - including your raw data, methods, protocols, software, hardware and more – which support your original research article in a Research Elements journal. Research Elements are open access, multidisciplinary, peer-reviewed journals which make the objects associated with your research more discoverable, trustworthy and promote replicability and reproducibility. As open access journals, there may be an Article Publishing Charge if your paper is accepted for publication. Find out more about the Research Elements journals at https://www.elsevier.com/authors/tools-and-resources/research-elements-journals?dgcid=ec_em_research_elements_email.

One Health values your contribution and I look forward to receiving your revised manuscript.

Kind regards,

Pabulo Rampelotto
Scientific Handling Editor
One Health

==

Please include the following items when submitting your revised manuscript:

- A rebuttal letter that responds to each point raised by the academic editor and reviewers. Specify exactly where changes were made in the text (e.g., line number).
- A marked-up copy of your manuscript that highlights changes made to the original version. Use yellow highlighting to indicate the revisions made in response to reviewers and editor comments. Please do not use any tracked changes.
- An unmarked version of your revised paper without yellow marks.
- Remove previous versions of the files. Keep only the most recent revised and uploaded version.
- To facilitate the review process, please follow standard manuscript form, with double-space as line spacing as well as line numbers. Also, number heading and subheadings, e.g. "1. Introduction", and so on.
- Authorship changes are not permitted at this stage. Please keep this in mind, as any attempt to alter authorship will result in the rejection of the paper.

Editor #:

• Highlights have more than 85 characters. They should be within the required limitation (85 characters, including spaces). Please use the "character count" feature in your word processor to check the length.

• The quality of the figures and graphical abstract provided is low and the figures are blurred. Please submit high-quality files and make sure they are in high resolution and are not blurry when zooming.

• Please submit a separate high-quality file for each Figure in PNG (or TIFF). Make sure they are in high resolution and are not blurry when zooming (just saving them with 300/600 dpi is not enough). Don't forget to properly name them as Figure 1, Figure 2, etc.

• In Discussion, explore the limitations of the study and discuss how to address them in future studies.

• In the manuscript, please provide more details regarding ethical approval, including name of the ethical committee/institution, protocol number, and data of approval. Also inform about participants written consent.

• 6 references were not validated by the system. Please check them and replace them with valid references from journals indexed in relevant databases (i.e. Scopus, PUBMED, Web of Science), if necessary.

• I noted the use of AI in the writing of your manuscript, so please include the following statement at the end of the manuscript (for more details, please see the journal instructions to the authors):

Declaration of generative AI and AI-assisted technologies in the writing process

During the preparation of this work the author(s) used [NAME TOOL / SERVICE] in order to improve readability and language. After using this tool/service, the authors reviewed and edited the content as needed and take full responsibility for the content of the published article.

Reviewer #1: This manuscript presents a comprehensive and valuable four-year genomic surveillance study of SARS-CoV-2 within a large university community. The longitudinal scope of the work is a significant strength, providing a detailed and rare perspective on the local evolution and transmission

dynamics of the virus in a semi-contained, high-contact setting. The central finding, that the university serves as a microcosm of global SARS-CoV-2 trends, albeit with a temporal lag and some unique local features-is well-supported by the data and is an important contribution to the field of public health and genomic epidemiology.

The methodology is robust, clearly described, and appropriately leverages established tools and databases (GISAID, Pangolin). The analyses are thorough, covering clade and lineage diversity, demographic distributions, and phylogenetic relationships. The comparison with global data effectively contextualizes the local findings.

The manuscript is generally well-written and the data are presented clearly in the figures and tables. This work underscores the critical role that academic institutions can play in national and global public health surveillance networks.

Major Comments:

1. Clade Subclassification Rationale and Utility: The authors introduce a new "clade subclassification" system to address limitations in the GISAID clade nomenclature, particularly for Omicron. While this is a thoughtful approach, its utility and impact on the study's conclusions need to be more clearly articulated. Suggestion: Please include a brief discussion or a supplementary figure demonstrating how this new classification provided insights that the standard GISAID/Pango lineage classification did not. How did it improve the temporal or epidemiological resolution, as claimed?
2. Interpretation of Demographic Findings: The manuscript reports higher lineage diversity in the undergraduate age group and notes the presence of sex-specific lineages. However, the interpretation of these findings is somewhat cautious. Suggestions: For the age finding, please discuss more explicitly whether the higher diversity is likely a true epidemiological signal of more diverse transmission networks or simply a reflection of the over-representation of this age group in the sample (74% of samples). A normalization or a more direct statistical comparison accounting for sample size disparity would strengthen this claim. For the sex-specific lineages, the speculation on "subtle demographic determinants" could be slightly refined. Please consider discussing alternative, perhaps more parsimonious, explanations, such as chance findings due to the large number of lineages analyzed or behavioral factors not captured in the data.
3. Phylogenetic Analysis Presentation: The phylogenetic tree (Figure 7) is a key component for illustrating evolutionary relationships. However, in its current form (a circular cladogram in the main figure), it is difficult to interpret specific details, such as the clustering of lineages and bootstrap support. Suggestion: Consider moving the detailed circular tree to the supplementary materials and replacing it in the main text with a simpler, more focused phylogenetic tree (e.g., a rectangular tree highlighting the major variant transitions like Alpha, Delta, Omicron). Alternatively, ensure that the main figure is of sufficiently high resolution and has clear labels for the key lineages discussed in the text.

Minor Comments:

1. Abstract and Highlights: The abstract and highlights are clear but very dense. Consider slightly streamlining them to enhance readability for a broad audience.
2. Figure 2D: The caption for Figure 2D mentions lineages highlighted in red, but the provided figure is in grayscale. Please ensure that the final version uses color or distinct patterns for clarity.
3. Data Availability Statement: The data availability statement indicates that metadata is available upon request. To align with best practices for reproducibility, I encourage the authors to deposit the relevant, de-identified metadata in a public repository where feasible, or to state this more explicitly in the manuscript.

Reviewer #2: See attached.

More information and support

FAQ: How do I revise my submission in Editorial Manager?

https://service.elsevier.com/app/answers/detail/a_id/28463/supporthub/publishing/

FAQ: How can I reset a forgotten password?
https://service.elsevier.com/app/answers/detail/a_id/28452/supporthub/publishing/
For further assistance, please visit our customer service site:
https://service.elsevier.com/app/home/supporthub/publishing/
Here you can search for solutions on a range of topics, find answers to frequently asked questions, and learn more about Editorial Manager via interactive tutorials. You can also talk 24/7 to our customer support team by phone and 24/7 by live chat and email

At Elsevier, we want to help all our authors to stay safe when publishing. Please be aware of fraudulent messages requesting money in return for the publication of your paper. If you are publishing open access with Elsevier, bear in mind that we will never request payment before the paper has been accepted. We have prepared some guidelines (https://www.elsevier.com/connect/authors-update/seven-top-tips-on-stopping-apc-scams) that you may find helpful, including a short video on Identifying fake acceptance letters (https://www.youtube.com/watch?v=o5l8thD9XtE). Please remember that you can contact Elsevier s Researcher Support team (https://service.elsevier.com/app/home/supporthub/publishing/) at any time if you have questions about your manuscript, and you can log into Editorial Manager to check the status of your manuscript (https://service.elsevier.com/app/answers/detail/a_id/29155/c/10530/supporthub/publishing/kw/status/).

#AU_ONEHLT#

To ensure this email reaches the intended recipient, please do not delete the above code

---

In compliance with data protection regulations, you may request that we remove your personal registration details at any time. (Remove my information/details). Please contact the publication office if you have any questions.

One Health

Microcosm of a pandemic: what four years of genomic surveillance in a university community reveal about SARS-CoV-2

--Manuscript Draft--

**Manuscript Number:**	ONEHLT-D-25-01096R1
**Article Type:**	Research Paper
**Section/Category:**	Public Health
**Keywords:**	SARS-CoV-2; genomic surveillance; clades; lineages; variants; Phylogenetics; demography; University community; global comparison
**Corresponding Author:**	Sankar Prasad Chaki, Ph.D<br>Texas A&M University College Station<br>College Station, Texas UNITED STATES OF AMERICA
**First Author:**	Sankar Prasad Chaki, Ph.D
**Order of Authors:**	Sankar Prasad Chaki, Ph.D
	Melissa M Kahl-McDonagh, Ph.D
	Benjamin W Neuman, Ph.D
	Loni A. Taylor, Ph.D, DVM
	Rebecca SB Fischer
	Blake M Hanson, Ph.D
	Marlisa S Hardy
	Jo Ann Culpepper
	Eric Boerwinkle, Ph.D
	Kurt A Zuelke, Ph.D
**Abstract:**	Objectives Although SARS-CoV-2 transmission is currently minimal, ongoing viral evolution continues to pose public health challenges. This study characterized the diversity, persistence, and demographic distribution of SARS-CoV-2 clades, lineages, and variants in a university community over four years and compared local patterns with global trends to better understand transmission dynamics and guide future surveillance and mitigation strategies. Methods We conducted four years of genomic surveillance of SARS-CoV-2 in a globally diverse university community by collecting samples from the university health services and at campus non-clinical testing sites. The sequencing data was classified into GISAID clades, Pango lineages and WHO variants, and compared with the Global Initiative on Sharing All Influenza Data (GISAID). Temporal analyses were performed to assess diversity, persistence, demographic distribution, and the dynamics of SARS-CoV-2 evolution within the community. Results Over four years, genomic surveillance in the university community identified multiple SARS-CoV-2 clades and hundreds of distinct lineages, capturing local viral evolution and transmission dynamics. Most lineages were transient, though some persisted over time. Many prevalent local lineages reflected global trends, and variant patterns broadly mirrored global waves. Lineage diversity was highest among undergraduate student age group, with similar distributions across sexes. Peak detection occurred during fall semesters. Phylogenetic analyses illustrated the emergence, spread, and persistence of lineages, highlighting the university as a microcosm of global SARS-CoV-2 evolution. Conclusions SARS-CoV-2 dynamics in the university community closely parallel global trends, with some temporal lag. Targeted genomic surveillance in semi-contained, high-contact settings offers real-time insights into viral evolution and informs early planning of effective public health strategies.
**Opposed Reviewers:**	
**Response to Reviewers:**	

Powered by Editorial Manager® and ProduXion Manager® from Aries Systems Corporation

GLOBAL HEALTH RESEARCH COMPLEX

TEXAS A&M UNIVERSITY

February 2, 2026

To
Dr. Aileen M. Marty
Editor-in-Chief, One Health
Florida International University Herbert Wertheim College of Medicine
Miami, Florida, United States of America

Dear Dr. Marty,

With great enthusiasm, we are submitting our revised manuscript entitled "Microcosm of a pandemic: what four years of genomic surveillance in a university community reveal about SARS-CoV-2" for consideration for publication in One Health. We are grateful for the detailed and insightful feedback from the reviewers and editor, which has helped us strengthen the manuscript.

We have revised the manuscript accordingly and provided a detailed, point-by-point response to address all comments. We believe the revisions have improved the work substantially, and we respectfully submit it for your further consideration.

Thank you for your time and consideration. We believe this manuscript will be a meaningful addition to One Health.

Sincerely,

Sankar Prasad Chaki, PhD
Research Scientist
Global Health Research Complex
Division of Research, Texas A&M University
Mail to: spchaki@tamu.edu

Response to Reviewer Comment
Chaki SP et. al.

Editor #

Comment 1: Highlights have more than 85 characters. They should be within the required limitation (85 characters, including spaces). Please use the "character count" feature in your word processor to check the length.

Answer: We apologize for the mistake and rectified the character limit in the highlights (**Page 2, Lines: 19, 22, 23**).

Comment 2: The quality of the figures and graphical abstract provided is low and the figures are blurred. Please submit high-quality files and make sure they are in high resolution and are not blurry when zooming.

Answer: Thank you for the feedback. High-resolution figures and an updated graphical abstract have now been uploaded. The revised files are submitted in high quality and remain clear without blurring when zoomed.

Comment 3: Please submit a separate high-quality file for each Figure in PNG (or TIFF). Make sure they are in high resolution and are not blurry when zooming (just saving them with 300/600 dpi is not enough). Don't forget to properly name them as Figure 1, Figure 2, etc.

Answer: Thank you for the comment. Separate high-resolution files for each figure have now been uploaded in the required format (TIFF). Each file has been checked to ensure clarity at zoom and is properly labeled as Figure 1, Figure 2, etc., according to the journal requirements.

Comment 4: In Discussion, explore the limitations of the study and discuss how to address them in future studies.

Answer: Thank you for the suggestion. We have now expanded the Discussion section to include the limitations of the study and provided corresponding recommendations for how these limitations could be addressed in future research (**Page 18, Line: 387 - 394**).

Comment 5: In the manuscript, please provide more details regarding ethical approval, including name of the ethical committee/institution, protocol number, and data of approval. Also inform about participants written consent.

Answer: Thank you for the comment. The manuscript has been updated to include additional details regarding ethical approval, including the name of the approving committee and institution. This study used publicly available data uploaded to and downloaded from GISAD for analysis (**Page 8, Line: 172-173**).

Comment 6. 6 references were not validated by the system. Please check them and replace them with valid references from journals indexed in relevant databases (i.e. Scopus, PUBMED, Web of Science), if necessary.

Answer: References were checked and found necessary. Although they are not indexed in the requested databases, majority of them represent original federal government documents and are essential primary sources for this study. Therefore, they have been retained. Reference 25 (Colwell RK, Coddington JA) newly added (**Page 21, Line: 495-496**).

Comment 7. I noted the use of AI in the writing of your manuscript, so please include the following statement at the end of the manuscript (for more details, please see the journal instructions to the authors): Declaration of generative AI and AI-assisted technologies in the writing process. During the preparation of this work the author(s) used [NAME TOOL / SERVICE] in order to improve readability and language. After using this tool/service, the authors reviewed and edited the content as needed and take full responsibility for the content of the published article.

Answer: Thank you for your comment. We appreciate the guidance and have now included the required statement in the manuscript (**Page 20, Line: 428-432)**.

Reviewer comments:

Reviewer #1

General comment: This manuscript presents a comprehensive and valuable four-year genomic surveillance study of SARS-CoV-2 within a large university community. The longitudinal scope of the work is a significant strength, providing a detailed and rare perspective on the local evolution and transmission dynamics of the virus in a semi-contained, high-contact setting. The central finding, that the university serves as a microcosm of global SARS-CoV-2 trends, albeit with a temporal lag and some unique local features-is well-supported by the data and is an important contribution to the field of public health and genomic epidemiology.
The methodology is robust, clearly described, and appropriately leverages established tools and databases (GISAID, Pangolin). The analyses are thorough, covering clade and lineage diversity, demographic distributions, and phylogenetic relationships. The comparison with global data effectively contextualizes the local findings.
The manuscript is generally well-written and the data are presented clearly in the figures and tables. This work underscores the critical role that academic institutions can play in national and global public health surveillance networks.

Answer: We thank the reviewer for their constructive and encouraging feedback. We are pleased that the study design, analyses, and presentation were viewed favorably.

Major Comments:

Comment 1: Clade Subclassification Rationale and Utility: The authors introduce a new "clade subclassification" system to address limitations in the GISAID clade nomenclature, particularly for Omicron. While this is a thoughtful approach, its utility and impact on the study's conclusions need to be more clearly articulated.

Suggestion: Please include a brief discussion or a supplementary figure demonstrating how this new classification provided insights that the standard GISAID/Pango lineage classification did not. How did it improve the temporal or epidemiological resolution, as claimed?

Answer 1: Thank you for the helpful comment. In response, we have added a brief explanation in the discussion clarifying the utility of the clade subclassification system and how it improves temporal and epidemiological resolution compared to the standard GISAID nomenclature (**Page 15, Line: 318-328**), and we strengthened this point by linking the data in Table 1 with Figure 1A.

Comment 2: Interpretation of Demographic Findings: The manuscript reports higher lineage diversity in the undergraduate age group and notes the presence of sex-specific lineages. However, the interpretation of these findings is somewhat cautious. Suggestions: For the age finding, please discuss more explicitly whether the higher diversity is likely a true epidemiological signal of more diverse transmission networks or simply a reflection of the over-representation of this age group in the sample (74% of samples). A normalization or a more direct statistical comparison accounting for sample size disparity would strengthen this claim. For the sex-specific lineages, the speculation on "subtle demographic determinants" could be

slightly refined. Please consider discussing alternative, perhaps more parsimonious, explanations, such as chance findings due to the large number of lineages analyzed or behavioral factors not captured in the data.

Answer 2: Thank you for the comment. To account for differences in sampling depth across age and sex groups and to distinguish true epidemiological patterns from sampling bias, we used the Chao1 estimator to correct lineage richness (**Page 8, Line: 166-170**), and the corresponding figure (Figure 4) and figure legend (**Page 25, Line: 572 and 575**) have been updated.

Comment 3: Phylogenetic Analysis Presentation: The phylogenetic tree (Figure 7) is a key component for illustrating evolutionary relationships. However, in its current form (a circular cladogram in the main figure), it is difficult to interpret specific details, such as the clustering of lineages and bootstrap support. Suggestion: Consider moving the detailed circular tree to the supplementary materials and replacing it in the main text with a simpler, more focused phylogenetic tree (e.g., a rectangular tree highlighting the major variant transitions like Alpha, Delta, Omicron). Alternatively, ensure that the main figure is of sufficiently high resolution and has clear labels for the key lineages discussed in the text.

Answer 3: Thank you for the comment. We have updated figure 7 to improve clarity by applying a color code to distinguish major variants (Alpha, Gamma, Delta, Omicron and Wuhan) making evolutionary relationships and major variant transitions easier to interpret.

Minor Comments:

Comment 1: Abstract and Highlights: The abstract and highlights are clear but very dense. Consider slightly streamlining them to enhance readability for a broad audience.

Answer 1: Abstract (**Page 2 & 3, Line: 25-43)** and highlights (**Page 2, Line: 19, 22, and 23)** have been streamlined for clarity and readability.

Comment 2: Figure 2D: The caption for Figure 2D mentions lineages highlighted in red, but the provided figure is in grayscale. Please ensure that the final version uses color or distinct patterns for clarity.

Answer 2: Figure 2D was updated with color to match the caption.

Comment 3: Data Availability Statement: The data availability statement indicates that metadata is available upon request. To align with best practices for reproducibility, I encourage the authors to deposit the relevant, de-identified metadata in a public repository where feasible, or to state this more explicitly in the manuscript.

Answer 3: We have updated the data availability statement to include a direct link to the de-identified metadata, which is publicly accessible at GISAID (**Page 20, Line: 425-427**). This ensures that all relevant data are available for reproducibility and further research.

Reviewer 2

General Comments: This is a comprehensive and well-executed longitudinal genomic surveillance study that captures SARS-CoV-2 diversity and transmission dynamics within a university community. The manuscript is timely, data-rich, and provides valuable insights into post-pandemic viral evolution. However, some minor revision would significantly enhance its scientific rigor and readability.

Answer: We thank the reviewer for the positive feedback and for recognizing the value of our study. We have addressed the suggested minor revisions to improve the scientific rigor and readability of the manuscript.

Minor Comments

Comment 1: The introduction could more explicitly articulate the study's unique contribution compared with prior SARS-CoV-2 genomic surveillance studies.

Answer 1: We thank the reviewer for this suggestion. The Introduction has been revised to clearly highlight our study's unique contributions to SARS-CoV-2 genomic surveillance (**Page 4, Line: 72-78)**.

Comment 2: Figures 2A, 2B, 2D, 3B, and 6A, as well as Figure 7 (where the color code should be clearly defined), would benefit from organizing the variants under their corresponding major lineages (e.g., Alpha, Beta, Delta, Omicron). Presenting numerous clades independently makes it difficult for readers to follow the evolutionary relationships. Grouping the clades within these well-recognized variant lineages and clearly defining color codes would significantly improve the figures' clarity, interpretability, and visual coherence.

Answer 2: We thank the reviewer for this valuable suggestion. We have revised Figures 2A, 2B, 2D, 3B, 6A, and 7 to group variants under their corresponding major lineages (e.g., Alpha, Gamma, Delta, Omicron) and have clearly defined the color codes. Corresponding figure legends were also updated (**Page 24, Line: 557, 558, 562, 563, 567, 568, 569, 584, 586, 587, 591, 592, and 593**).

We sincerely thank the editor and reviewer for their thoughtful and encouraging feedback. We are pleased that the longitudinal design, analytical approach, and clarity of data presentation were well-received. We also appreciate the recognition of the value of this work in contributing to genomic epidemiology and public health surveillance.

Date: Feb 18, 2026
To: "Sankar Prasad Chaki" spchaki@tamu.edu
cc: p.rampelotto@elsevier.com
From: "One Health" support@elsevier.com
Subject: Decision on submission to One Health

Manuscript Number: ONEHLT-D-25-01096R1

Microcosm of a pandemic: what four years of genomic surveillance in a university community reveal about SARS-CoV-2

Dear Dr. Chaki,

Thank you for submitting the revised version of your manuscript. While you have addressed the reviewers' comments and suggestions, we find that the manuscript currently does not align with the standards required for publication in One Health, as it is out of scope for the journal. For more details, please access Aims and scope - One Health | ScienceDirect.com by Elsevier

Please note that our editorial assessment follows the publisher's guidelines to respect your journal choice and to ensure an impartial peer review process. Based on the reviewers' feedback and the journal's criteria for publication, we evaluate the suitability of the revised manuscript. If the manuscript is deemed suitable for publication but falls outside the scope of the current journal, we can offer to transfer it to other appropriate journals within our portfolio. This approach allows more authors to benefit from valuable feedback and revision opportunities, ultimately increasing their chances of successful publication either in the journal or through the transfer process. For further context on our approach, you may find the article Why Peer Review More? helpful: https://www.elsevier.com/connect/why-peer-review-more.

I understand that this outcome will be disappointing, but we are keen to help you publish your manuscript in a timely manner. Given the topic of your manuscript, our editorial assessment of your work, and the feedback from reviewers, **I am happy to offer you the opportunity to publish it without further peer review** in our partner journal:

• Next Research, the peer-reviewed multidisciplinary journal in the Next family of journals, invites authors to publish original and scientifically rigorous research across all disciplines, accepting both theoretical and experimental work. As a broad-scope journal, our primary criterion for publication is the scientific accuracy and ethical integrity of the work, rather than its perceived impact. Submissions to Next Research are initially exclusively by transfer invitation; our dedicated team ensures a seamless and supportive publication experience from start to finish. [More information: https://www.sciencedirect.com/journal/next-research]

If you take up this offer and fully address the comments of the reviewers in your revised manuscript, your submission will be quickly accepted.

To take advantage of this conditional acceptance offer, simply click the link at the end of this letter. You will then have 90 days to finalise your submission. During this stage, please upload a tracked-change revision of your manuscript as well as a comprehensive "Response to Reviewers" confirming that the required revisions have been implemented. Just in case the handling editor of the suggested journal is not fully satisfied with your revisions, the editor may invite previous or new reviewers to evaluate the adequacy of your revised manuscript.

Alternatively, you may wish to submit your manuscript to the following journals for consideration. Please note [illegible] your paper will proceed through the standard editorial process.

- Virus Research https://www.sciencedirect.com/journal/virus-research

To transfer your paper, please click the link below by Jun 28, 2026. You will also have 90 days to finalise your submission at your preferred journal.

****

If you do not wish to transfer your manuscript, click here: ********

Kind regards,

Pabulo Rampelotto
Scientific Handling Editor
One Health

More information and support

* When the transfer of your manuscript to the alternative journal is complete (usually within 48 hours) you will receive an email containing further instructions on how to complete your submission.

* You will be able to revise your manuscript prior to resubmission. When you submit your revised manuscript, please ensure you include a cover letter detailing all changes to the manuscript, as well as a "Response to Reviewers" containing all reviewer comments and a response to each comment.

* Once your submission is complete it will be considered by the Editor(s) of the alternative journal. While I cannot guarantee publication in the alternative journal, it is expected your manuscript will be accepted if the comments below are addressed.

You will find more information about Elsevier's Article Transfer Service here: www.elsevier.com/authors/article-transfer-service.

You will find information relevant for you as an author on Elsevier's Author Hub: https://www.elsevier.com/authors

FAQ: How can I reset a forgotten password?
https://service.elsevier.com/app/answers/detail/a_id/28452/supporthub/publishing/
For further assistance, please visit our customer service site:
https://service.elsevier.com/app/home/supporthub/publishing/
Here you can search for solutions on a range of topics, find answers to frequently asked questions, and learn more about Editorial Manager via interactive tutorials. You can also talk 24/7 to our customer support team by phone and 24/7 by live chat and email

#AU_ONEHLT#

To ensure this email reaches the intended recipient, please do not delete the above code.

In compliance with data protection regulations, you may request that we remove your personal registration details at any time. (Remove my information/details). Please contact the publication office if you have any questions.

Re: Spectrum00860-26R1 (Longitudinal genomic surveillance of SARS-CoV-2 in a university microcosm reflects global evolutionary trends)

Dear Dr. Sankar Prasad Chaki:

Thank you again for sharing your longitudinal surveillance of SARS-CoV-2 variants in a university.

Your manuscript has been accepted, and I am forwarding it to the ASM production staff for publication. Your paper will first be checked to make sure all elements meet the technical requirements. ASM staff will contact you if anything needs to be revised before copyediting and production can begin. Otherwise, you will be notified when your proofs are ready to be viewed.

Data Availability: ASM policy requires that data be available to the public upon online posting of the article, so please verify all links to sequence records, if present, and make sure that each number retrieves the full record of the data. If a new accession number is not linked or a link is broken, provide production staff with the correct URL for the record. If the accession numbers for new data are not publicly accessible before the expected online posting of the article, publication may be delayed; please contact ASM production staff immediately with the expected release date.

Publication Fees: For information on publication fees and which article types have charges, please visit our website. We have partnered with Copyright Clearance Center (CCC) to collect author charges. If fees apply to your paper, you will receive a message from no-reply@copyright.com with further instructions. For questions related to paying charges through RightsLink, please contact CCC at ASM_Support@copyright.com or toll free at +1-877-622-5543. CCC makes every attempt to respond to all emails within 24 hours.

ASM Membership: Corresponding authors may join or renew ASM membership to obtain discounts on publication fees. Need to upgrade your membership level? Please contact Customer Service at Service@asmusa.org.

Embargo Policy: A press release may be issued as soon as the manuscript is posted on the Spectrum Latest Articles webpage. The corresponding author will receive an email with the subject line "ASM Journals Author Services Notification" when the article is available online.

The ASM Journals program strives for constant improvement in our submission and publication process. Please tell us how we can improve your experience by taking this quick Author Survey.

Thank you for submitting your paper to Spectrum.

Sincerely,
JJ Miranda
Editor
Microbiology Spectrum
